# Salt-induced subcellular kinase relocation and seedling susceptibility caused by overexpression of *Medicago* SIMKK in *Arabidopsis*

**DOI:** 10.1093/jxb/eru115

**Published:** 2014-03-19

**Authors:** Miroslav Ovečka, Tomáš Takáč, George Komis, Pavol Vadovič, Slávka Bekešová, Anna Doskočilová, Veronika Smékalová, Ivan Luptovčiak, Olga Šamajová, Alois Schweighofer, Irute Meskiene, Claudia Jonak, Pavel Křenek, Irene Lichtscheidl, Ľudovít Škultéty, Heribert Hirt, Jozef Šamaj

**Affiliations:** ^1^Centre of the Region Haná for Biotechnological and Agricultural Research, Department of Cell Biology, Faculty of Science, Palacký University, Šlechtitelů 11, 783 71 Olomouc, Czech Republic; ^2^Max F. Perutz Laboratories, Vienna Biocenter, University of Vienna, Dr Bohr-Gasse 9, A-1030 Vienna, Austria; ^3^Gregor Mendel Institute of Molecular Plant Biology GmbH, Dr Bohr-Gasse 3, A-1030 Vienna, Austria; ^4^Institution of Cell Imaging and Ultrastructure Research, University of Vienna, Althanstrasse 14, A-1090 Vienna, Austria; ^5^Department of Rickettsiology, Institute of Virology, Slovak Academy of Sciences, Dubravska cesta 9, Bratislava, 845 05, Slovakia; ^6^Unité de Recherche en Genomique Végétale, Université d’Evry-Val-d’essone, 2, rue Gaston Crémieux, F-91057 Evry, France

**Keywords:** *Arabidopsis*, MAPK, *Medicago*, proteomics, salt stress, SIMK, SIMKK, subcellular relocation.

## Abstract

Dual-specificity mitogen-activated protein kinases kinases (MAPKKs) are the immediate upstream activators of MAPKs. They simultaneously phosphorylate the TXY motif within the activation loop of MAPKs, allowing them to interact with and regulate multiple substrates. Often, the activation of MAPKs triggers their nuclear translocation. However, the spatiotemporal dynamics and the physiological consequences of the activation of MAPKs, particularly in plants, are still poorly understood. Here, we studied the activation and localization of the *Medicago sativa* stress-induced MAPKK (SIMKK)–SIMK module after salt stress. In the inactive state, SIMKK and SIMK co-localized in the cytoplasm and in the nucleus. Upon salt stress, however, a substantial part of the nuclear pool of both SIMKK and SIMK relocated to cytoplasmic compartments. The course of nucleocytoplasmic shuttling of SIMK correlated temporally with the dual phosphorylation of the pTEpY motif. SIMKK function was further studied in *Arabidopsis* plants overexpressing SIMKK–yellow fluorescent protein (YFP) fusions. SIMKK–YFP plants showed enhanced activation of *Arabidopsis* MPK3 and MPK6 kinases upon salt treatment and exhibited high sensitivity against salt stress at the seedling stage, although they were salt insensitive during seed germination. Proteomic analysis of SIMKK–YFP overexpressors indicated the differential regulation of proteins directly or indirectly involved in salt stress responses. These proteins included catalase, peroxiredoxin, glutathione *S*-transferase, nucleoside diphosphate kinase 1, endoplasmic reticulum luminal-binding protein 2, and finally plasma membrane aquaporins. In conclusion, *Arabidopsis* seedlings overexpressing SIMKK–YFP exhibited higher salt sensitivity consistent with their proteome composition and with the presumptive MPK3/MPK6 hijacking of the salt response pathway.

## Summary

This study revealed activation-dependent and coordinated relocation of *Medicago sativa* SIMKK and SIMK after salt stress. *Arabidopsis* seedlings stably overexpressing YFP-tagged SIMKK showed altered salt sensitivity and proteome changes.

## Introduction

Mitogen-activated protein kinases (MAPKs) perceive and transduce various signals affecting plant life. MAPK pathways are organized into three-tiered modules composed of a MAPK kinase kinase (MAPKKK) activating a dual-specificity Ser/Thr and Tyr MAPK kinase (MAPKK), resulting in the dual phosphorylation and activation of a Ser/Thr MAPK (Jonak *et al.*, 2002; Rodriguez *et al.*, 2010). MAPKKs activate MAPKs by simultaneous phosphorylation of TEY or TDY signature motifs, within the kinase activity loop (Kiegerl *et al.*, 2000; Calderini *et al.*, 2001; Keshet and Seger, 2010). In *Arabidopsis thaliana* L., 20 different MAPK pathways have been identified in the complete annotated genome (MAPK Group, 2002; Colcombet and Hirt, 2008; Dóczi *et al.*, 2012; Janitza *et al.*, 2012).

Plant MAPKs can be activated by multiple abiotic stimuli such as wounding, drought, cold, and salinity (Rodriguez *et al.*, 2010; Sinha *et al.*, 2011; Šamajová *et al.*, 2013*a*) and biotic stresses such as diverse pathogens, pathogen-derived toxins, and microbe-associated molecular patterns or MAMPs (Pitzschke *et al.*, 2009; Rasmussen *et al.*, 2012). In response to environmental and developmental cues, plant MAPKs mediate hormonal responses, cell-cycle regulation, and development (Komis *et al.*, 2011; Sasabe and Machida, 2012; Šamajová *et al.*, 2013*a*). In this respect, MAPKs phosphorylate, and thereby regulate, diverse intracellular targets including other protein kinases, nuclear transcription factors, cytoskeletal components, and proteins involved in vesicular trafficking in eukaryotic cells (Cargnello and Roux, 2011; Komis *et al.*, 2011; Šamajová *et al.*, 2013*b*). Therefore, MAPKs represent versatile transducers of plant signalling.

In model systems such as yeast and mammalian cells, MAPK modules undergo dynamic compartmentalization. The prototypical extracellular signal-regulated kinase 1 (ERK1) exhibits conditional nucleocytoplasmic shuttling in response to mitogen stimulation (Volmat *et al.*, 2001). Cellular localization of plant MAPK cascades and their molecular interactions are much less understood. Previous reports in dividing plant suspension cells showed relocation of MAPKs during biotic stress (Ligterink *et al.*, 1997; Lee *et al.*, 2004). The differential subcellular localization of MAPKs was particularly described during developmental processes including cytokinesis and root hair formation (Calderini *et al.*, 1998; Bögre *et al.*, 1999; Coronado *et al.*, 2002; Šamaj *et al.*, 2002; Beck *et al.*, 2010, 2011; Kosetsu *et al.*, 2010; Müller *et al.*, 2010). Co-localization of MAPKs with their upstream MAPKKs and their joint translocation to certain cellular compartments has been documented only rarely, including the ethylene-induced simultaneous nuclear translocation of MKK9–MPK3/6 (Yoo *et al.*, 2008). However, insight into the regulation and function of the subcellular localization of MAPK pathways is lacking in plants.

In *Medicago sativa*, SIMK (stress-induced MAPK) was identified as a salt stress- and elicitor-induced MAPK (Munnik *et al.*, 1999; Cardinale *et al.*, 2000). Yeast two-hybrid screening and *in vitro* and *in vivo* activation studies identified SIMK kinase (SIMKK) as the upstream activator of SIMK. SIMKK was shown to activate SIMK in response to salt stress (Kiegerl *et al.*, 2000; Cardinale *et al.*, 2002). This study aims to provide insight into the subcellular localization of SIMKK and SIMK, revealing that a significant part of the nuclear pool of the two kinases become relocated to the cytoplasm upon activation by salt stress. Interestingly, upon salt activation, SIMKK and SIMK are concentrated in unknown subcellular cytoplasmic punctate structures that require further investigation. Finally, we have presented proteomic, biochemical, and phenotypic characterization of *Arabidopsis* plants overexpressing SIMKK. These plants contained altered levels of proteins involved in salt and oxidative stress, higher activity levels of MPK6 and MPK3 after short salt treatment, and they were more susceptible to long-term salt stress.

## Materials and methods

### Plant material and treatments

Seeds of *M. sativa* L. cv. Europe were placed on moist filter paper in Petri dishes and germinated in culture chambers in darkness at 25 °C. Three-day-old seedlings were selected for salt treatments, immunoblotting, and immunolocalization experiments. Seeds of wild-type *A. thaliana* L., cv. Columbia and stably transformed lines were germinated and grown on agar or Phytagel plates containing half-strength Murashige and Skoog medium under standard culture conditions. Protoplasts were isolated from *Arabidopsis* suspension cultures as described previously (Kiegerl *et al.*, 2000; Cardinale *et al.*, 2002). Transiently transformed protoplasts, *M. sativa* roots, and seedlings of stably transformed *Arabidopsis* lines were treated with 250mM NaCl diluted in the culture medium. Stably transformed *Arabidopsis* plants with fluorescently tagged SIMKK constructs were also used for MAPK salt activation (treatment with 250mM NaCl for 10 and 30min) and for long-term salt treatments with 100mM NaCl. Images of the Petri dishes were taken 14 d after the transfer of 5-d-old plants to salt-containing medium. For germination tests, seeds of control and stably transformed *Arabidopsis* lines were sown on control medium or medium containing 100mM NaCl, kept at 4 °C for 48h, and transferred to a growing chamber under standard culture conditions. Germination rate was evaluated under a stereomicroscope on day 1, 2, and 3 after transfer to the chamber. Each experiment was repeated in five biological repeats.

### Vector constructs

Both SIMKK and SIMK were tagged on their C terminus with reporter genes encoding cyan fluorescent protein (*CFP*), yellow fluorescent protein (*YFP*) or the haemagglutinin (HA) epitope. All tags were subcloned as *Not*I/*Not*I fragments. Reading frames of the *SIMKK* and *SIMK* genes tagged with *CFP*, *YFP*, or HA were cloned into pSH9 or PRT 100 vectors using *Hin*dIII and *Pst*I under the control of the cauliflower mosaic virus (CaMV) 35S promoter. For stable expression in *Arabidopsis* plants, expression cassettes with *SIMKK–CFP/YFP* constructs under the control of the CaMV 35S promoter were cloned into the binary vector P-Green II.

### Plant transformation

*Arabidopsis* plants were stably transformed with *SIMKK–CFP/YFP* constructs using the standard floral-dip method (Clough and Bent, 1998). Protoplasts were transformed with *SIMKK–YFP/CFP* and *SIMK–YFP* constructs using a polyethylene glycol method as described previously (Kiegerl *et al.*, 2000; Cardinale *et al.*, 2002). *M. sativa* root cells were transiently transformed with *SIMKK–YFP* using the gene gun method according to the manufacturer’s instructions (Helios gene gun system; Bio-Rad) and the fluorescence of individually transformed cells was observed the next day.

### Antibodies and immunoblotting

Both protein A- and immunoaffinity-purified polyclonal antibodies N103 (recognizing the CTDFMpTEpYVVTRWC peptide of SIMK) and M23 (recognizing the C-terminal heptapeptide FNPEYQQ of SIMK; Cardinale *et al.*, 2000) were tested on root and protoplast extracts as described by Kiegerl *et al.* (2000) and Šamaj *et al.* (2002). For protein extraction, roots were homogenized in ice-cold extraction buffer [50mM Tris/HCl, pH 8, 150mM NaCl, 1% (v/v) NP-40, and 0.1% (w/v) SDS] and the protein content was measured using a Bradford assay. Protein extracts were separated by SDS-PAGE (MINI-Protean II cell system; Bio-Rad) and blotted onto nitrocellulose membrane. The membrane was blocked with 3% (w/v) bovine serum albumin and 3% (w/v) non-fat dried milk powder in Tris-buffered saline (TBS: 100mM Tris/HCl, pH 7.4, 1.5mM NaCl) for 1h, and subsequently incubated with a primary anti-green fluorescent protein (GFP) antibody (Sigma), diluted 1:1000 in TBS-T [TBS plus 0.1% (v/v) Tween 20] containing 1% (w/v) bovine serum albumin at room temperature for 1.5h.

For MAPK activation study, proteins were extracted from liquid nitrogen powders of roots or aerial parts of *Arabidopsis* plants before and after salt treatment (250mM for 30min) in 2 vols of RIPA buffer [50mM Tris/HCl, pH 7.4, 150mM KCl, 5mM EGTA, 0.5% (w/v) sodium deoxycholate, 0.1% (v/v) Triton X-100, 0.1% (w/v) SDS, phosphatase and protease inhibitors (PhosStop™ and EDTA-free Complete™; both from Roche)]. Extracts were then cleared (13 000*g*, 10min, 4 °C) and the supernatant was used as a source of total protein after quantitative determination by a Bradford assay. If the quantity of starting material was low, then aliquots of the supernatant were precipitated with 4 vols of anhydrous acetone (overnight at –20 °C), pelleted (13 000*g*, 10min, 4 °C), resuspended in 1/10vol. of rehydration buffer [8M urea, 2M thiourea, 2% (w/v) CHAPS], mixed proportionally with 4× Laemmli sample buffer and separated by 7.5% SDS-PAGE without heating. If the quantity of starting material was sufficient, then it was mixed proportionally with 4× Laemmli sample buffer and heat denatured before separating by 7.5% SDS-PAGE. After transfer to polyvinylidene difluoride membranes and Ponceau S visualization of the bands, the corresponding lanes were cut into strips and probed with anti-pTEpY phospho-p44/42 MAPK (Erk1/2,Thr202/Tyr204) (Cell Signalling Technology) diluted 1:1000, anti-MPK6 (Sigma, dilution 1:15 000), and anti-MPK3 (Sigma, dilution 1:4000) antibodies. For enhanced chemiluminescent detection, the strips were manually aligned under a ChemiDoc^TM^ MP Imaging System (Bio-Rad Laboratories) and exposed simultaneously.

### Immunokinase activity assays

Plant material was snap frozen in liquid nitrogen and extracted using extraction buffer (25mM Tris/HCl, pH 7.8, 10mM MgCl_2_, 15mM EGTA, 75mM NaCl, 1mM dithiothreitol, 1mM NaF, 0.5mM Na_3_VO_4_, 15mM β-glycerophosphate, 15mM 4-nitrophenylphosphate-bis[tris(hydroxymethyl)aminomethane], 0.1% (v/v) Tween 20, 0.5mM PMSF, 5 µg ml^–1^ of leupeptin and 5 µg ml^–1^ of aprotinin). Cell extracts containing 100 µg of total protein were immunoprecipitated overnight (4 °C) with 5 µg of protein A-purified SIMK antibody (Šamaj *et al.*, 2002). The immunoprecipitated kinase was washed three times with wash buffer [50mM Tris/HCl, pH 7.4, 250mM NaCl, 5mM EGTA, 0.1% (v/v) Tween 20, 5mM NaF] and once with kinase buffer (20mM HEPES, pH 7.4, 15mM MgCl_2_, 5mM EGTA, 1mM dithiothreitol). Kinase reactions were performed as described previously (Šamaj *et al.*, 2002).

### Fluorescence recovery after photobleaching (FRAP)

FRAP analysis of YFP-tagged SIMKK was performed employing a Leica TCS4D system using a 514nm Argon laser line for photobleaching of YFP. For quantitative analysis of FRAP experiments, the recovery of fluorescence was determined using Leica TCS4D software. Briefly, nuclei were selected as regions of interest with the zoom function and these areas were repeatedly bleached at full laser power so that the post-bleach fluorescence value represented maximally 10% of the original pre-bleach fluorescence value. The pre-bleach/post-bleach fluorescence and fluorescence recovery rates were recorded every 10 seconds using a time-lapse function. Fluorescence intensities were expressed in arbitrary units and normalized to absolute fluorescence intensities in the nuclei before bleaching. Corresponding half-time values of signal recovery and the portion of immobile protein fractions were calculated from FRAP data. Data from 12 regions of interest were exported to Microsoft Excel software, averaged and plotted as means±standard deviation.

### Immunolocalization of inactive and active SIMK and microscopy

For double immunolocalizations, the N103 and M23 antibodies were directly coupled to Alexa Fluor 488 (green fluorescence) and 568 (red fluorescence), with a commercial protein labelling kit (Invitrogen, Leiden, The Netherlands). Immunolocalization was performed on sections from Steedman’s wax embedded material as described previously (Šamaj *et al.*, 2002). Images of CFP/YFP/GFP-tagged SIMK and SIMKK in living cells as well as immunolabelled SIMK in fixed cells were acquired with an Axioplan 2 (Zeiss, Oberkochen, Germany) laser-scanning confocal microscope (Zeiss LSM710; Carl Zeiss, Jena, Germany) and Leica TCS4D (Leica, Mannheim, Germany), or a high-speed confocal microscope UltraVIEW life cell imager (PerkinElmer Life Sciences, Boston, USA) equipped with a spinning disc. Images were processed using Adobe Photoshop. Microfluorimetric image analysis of the immunofluorescence intensities of labelling in root cells was determined from Steedman’s wax sections according to Šamaj *et al*. (2002).

### Protein extraction for proteomics and trypsin digestion

Roots of 14-d-old transgenic *Arabidopsis* seedlings (in five independent biological replicates) carrying the SIMKK–YFP construct (line Y11) were used for proteomic analysis. The preparation of trypsin-digested extracts was performed as described by Takáč *et al*. (2011). Briefly, roots were homogenized in liquid nitrogen using a mortar and pestle and proteins were extracted by phenol extraction followed by successive methanolic ammonium acetate and acetone precipitation (Hurkman and Tanaka, 1986). The precipitates were dissolved in 6M urea and in total 100 µg of protein was reduced and alkylated with dithiothreitol and iodoacetamide, respectively. Proteins were digested with 20 μl of trypsin (0.1 μg μl^–1^) at 37 °C overnight. Digestion was stopped by the addition of 4 μl of 1% (v/v) formic acid. The peptides were desalted using SEP PAK light C18 columns (Waters, UK) according to manufacturer’s instructions.

### Liquid chromatography-electrospray ionization/elevated energy mass spectrometry (LC-ESI/MS^E^) analysis

Mass spectrometric analysis and quantification including one-dimensional nanoACQUITY ultraperformance liquid chromatography (UPLC) was performed according to Uváčková *et al*. (2013) with minor modifications. First, the trypsin-digested extracts were concentrated under vacuum to 10 μl. The final volume was then adjusted to 50 μl by adding 5% (v/v) acetonitrile in 0.1% (v/v) formic acid. Each sample was spiked with pre-digested bovine haemoglobin (prepared by digesting of HBA UNIPROT P01966) internal standard (Waters 186002327, UK) at a level of 1 pmol per 2 μl injection (2 μg of proteins). For sample analysis, 2 μg protein aliquots were analysed using a nanoACQUITY UPLC system (Waters) coupled to a Premier quadrupole time-of-flight (Q-TOF) mass spectrometer (Waters). The peptide mixture was injected onto a reverse-phase column (nanoACQUITY UPLC column BEH 130 C18, 75 μm×150mm, 1.7 μm particle size) and an acetonitrile gradient (10–50% acetonitrile containing 0.1% formic acid for 60min) at a flow rate of 350 nl min^–1^ was employed to elute the peptides into the Q-TOF. The column was connected to PicoTip emitters (New Objective, USA) mounted into the nanospray source of the Q-TOF Premier. A nano-electrospray voltage of 3.5kV was applied, with the source temperature set to 70 °C. The spectral acquisition scan rate was 1 s with a 0.05 s interscan delay. A novel multiplex MS^E^ approach, in which MS data were collected in an alternating low-energy (MS) and elevated energy (MS^E^) mode, was used for protein identification (Plumb *et al.*, 2006; Li *et al.*, 2009). For MS^E^ quantification, the average MS signal response from bovine haemoglobin was used to determine the universal signal response factor (counts per mol of protein). This information was then used to determine the concentrations for each of the target proteins by dividing the MS by the universal signal response factor. In the low-energy MS mode, data were collected at a constant collision energy of 2eV. In the elevated energy MS^E^ mode, the collision energy was ramped from 25 to 38eV during each integration. During data acquisition, the quadrupole analyser was not mass selective but operated in the radiofrequency-only mode. Thus, all ions were passed to the TOF analyser. Glu-1-fibrinopeptide B at a concentration of 1 pmol ml^–1^ was infused via the source at a flow rate of 500 nl min^–1^ and sampled every 30 s as the external mass calibrant.

### Data processing and database search

The MS^E^ data were processed using the ProteinLynx Global Server v.2.4 (PLGS 2.4; Waters), which provides background subtraction, smoothing, centroiding, and deisotoping. All data were then lock-spray calibrated against Glu-1-fibrinopeptide B using data collected from the reference line during acquisition and charge-state reduced to produce precursor monoisotopic mass lists as well as associated product ion mass lists. Each processed file was searched against the non-redundant *A. thaliana* UniProt database downloaded on 2 April 2013 (31 821 entries) with the addition of internal standard HBA_BOVIN haemoglobin subunit α sequence (Waters: 186002327; NCBI P01966) using the search algorithm within the PLGS 2.4. The initial correlation of a precursor (MS) and possible fragment ions (MS/MS) was achieved by means of time alignment. The elution profile of precursor masses from the LC component was used to deconvolute the mass of fragments from many precursor ions. Default search parameters were applied including the ‘automatic’ setting for mass accuracy (10 ppm for precursor ions and 15 ppm for product ions), a minimum of one peptide match per protein, a minimum of three consecutive product ion matches per protein, and a minimum of seven total product ion matches per protein. The maximum false-positive rate against the randomized forward database was set at 4%, and the protein quantification functionality was enabled using the internal standard (1 pmol of HBA_BOVIN haemoglobin subunit α). Only one missed tryptic cleavage site was allowed during the search. A fixed carbamidomethyl-Cys modification was used, in addition to the following variable modifications: deamidation of Asn and Gln, oxidation of Met, and dehydration of Ser and Thr. In order to determine protein quantities, the combined intensity of the multiply charged ions for the three most abundant tryptic peptides of a quantitatively added internal standard was compared with the observed response for any identified protein in a complex mixture. One-way analysis of variance statistical analysis was carried out to identify statistically significant (*P*<0.05) differences in protein amount.

## Results

In order to study the subcellular localization of SIMKK and its downstream target SIMK, we tagged these proteins with spectral variants of GFP (CFP and YFP) and expressed the gene constructs under the control of a CaMV 35S promoter. Individual or tandem expression was studied transiently in protoplasts isolated from *A. thaliana* suspension cells or in *M. sativa* roots. Expression after stable transformation was studied in *Arabidopsis* plants.

### Salt stress triggers nuclear export of YFP-tagged SIMKK and its association with cytoplasmic compartments *in vivo*

**Fig. 1.**
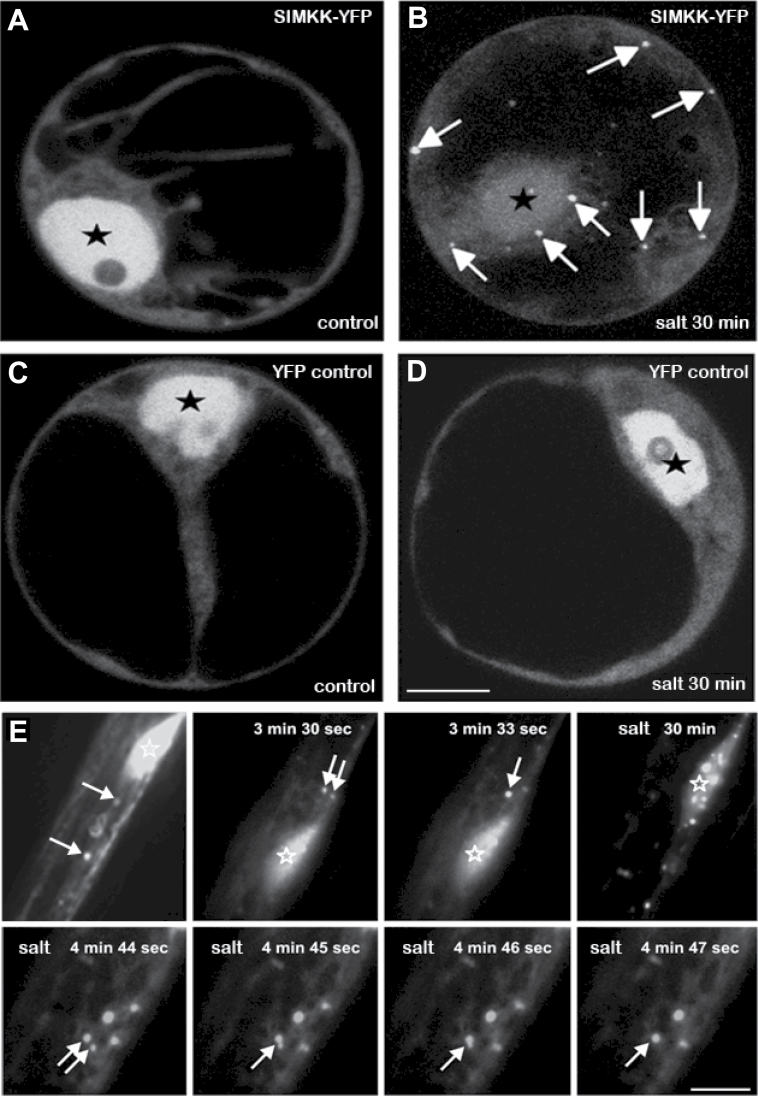
*In vivo* localization of YFP-tagged SIMKK in transiently transformed *Arabidopsis* protoplasts and in *M. sativa* roots. (A) SIMKK–YFP was located predominantly in the nucleus, but lower levels could also be detected in the cytoplasm of control protoplasts. (B) SIMKK–YFP relocates to cytoplasmic punctate compartments (indicated by arrows) in protoplasts treated with 250mM salt for 30min. (C, D) No relocation could be observed in salt-treated protoplasts transformed with control non-fused YFP. (E) In elongating epidermal root cell of *M. sativa*, SIMKK–YFP was located predominantly in the nucleus and partly in cytoplasmic motile compartments. Additional salt stress (250mM NaCl for 30min) caused accumulation of SIMKK–YFP in patches localized around nucleus. Arrows indicate some fast-moving and sometimes fusing cytoplasmic vesicular organelles with associated SIMKK–YFP. Figures represent individual frames (with indicated time points) from Supplementary Video S1 available at *JXB* online. Stars indicate nuclei. Bar, 7 μm (A–D); 10 μm (E).

In non-stressed transiently transformed *Arabidopsis* protoplasts, YFP-tagged SIMKK was localized predominantly in the nucleus and in the cytoplasm (Fig. 1A). Salt treatment (10–30min) reduced the nuclear content of SIMKK–YFP, whilst it relocated, at least partially, to cytoplasmic punctate compartments (Fig. 1B). No association of YFP alone (used as a control) with cytoplasmic compartments was observed under the same conditions of salt stress. In both control and salt-treated protoplasts, YFP alone remained predominantly in the nucleus (Fig. 1C, D). Thus, *in vivo* localization of YFP-tagged SIMKK in *Arabidopsis* protoplasts revealed salt stress-induced relocation of the kinase and its specific association with punctate compartments in the cytoplasm.

Localization of YFP-tagged SIMKK into stress-induced cytoplasmic compartments and their dynamic mobility was also studied in non-dividing elongated epidermal root cells of *M. sativa* following transient transformation. These cytoplasmic structures representing vesicular organelles were highly motile and moved with different velocities independently of cytoplasmic streaming, whilst they also accumulated in larger patches around the nucleus (Fig. 1E). Salt treatment of these cells significantly enhanced localization of SIMKK–YFP to cytoplasmic compartments occasionally fusing to each other (Fig. 1E; Supplementary Video S1 available at *JXB* online).

### Persistent salt-induced activation of SIMK, a downstream target of the SIMKK, in intact roots of *M. sativa*

**Fig. 2.**
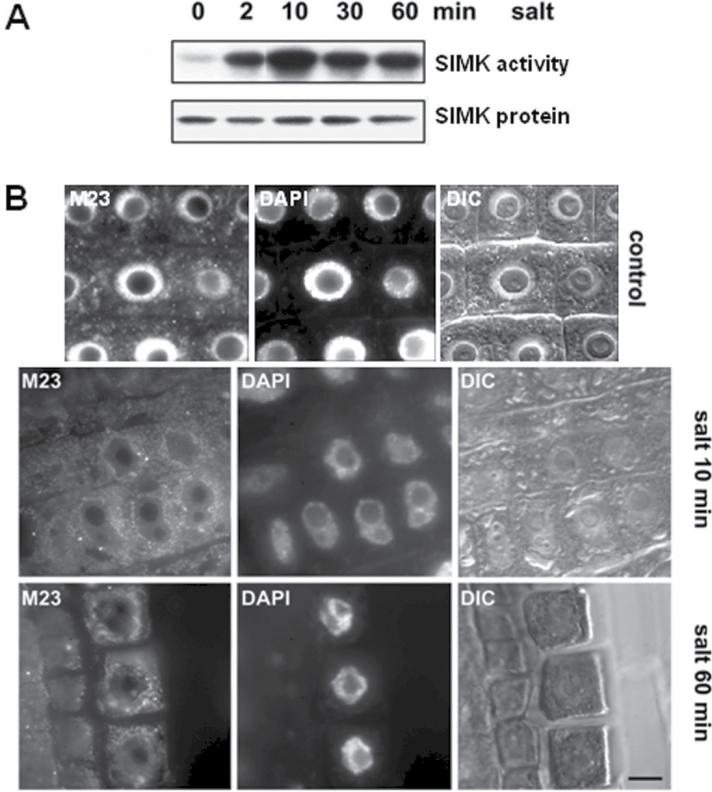
Salt-dependent activation and localization of SIMK in *M. sativa* roots. (A) Immunokinase analysis of time-dependent, persistent salt-induced activation of SIMK in intact *M. sativa* roots. Upper panel: *M. sativa* roots treated with 250mM NaCl for the indicated times. Root cell extracts were immunoprecipitated with protein A-purified SIMK-specific antibody M23. Kinase reactions were performed using MBP as a substrate. Lower panel: the same crude extracts from salt-treated roots were subjected to immunoblotting with the M23 antibody in order to check SIMK protein levels. (B) Immunolocalization of SIMK with M23 antibody in control and salt-treated (250mM NaCl) non-dividing cells of intact *M. sativa* roots. SIMK was abundant in the nuclei of control cells, whilst it relocated to punctate cytoplasmic compartments in salt-treated root cells. With increasing time (60min) of salt treatment, these cytoplasmic structures coalesced into larger patches that accumulated around the nuclei. Note the change in shape of the nuclei due to NaCl-induced plasmolysis. Images in the middle panel show nuclear DAPI staining. DIC, differential interference contrast. Bar, 5 μm (B).

The concentration and time-dependent salt-induced activation of SIMK, which is a direct target of SIMKK (Kiegerl *et al.*, 2000), was characterized in intact *M. sativa* roots, which are the organs typically affected by soil salinity. Within 2min, salt induced the activation of SIMK which persisted for at least 1h (Fig. 2A, upper panel) without affecting SIMK protein levels (Fig. 2A, lower panel). Thus, salt-induced SIMK activation occurred by a post-translational mechanism. These data showed that salt stress rapidly activates SIMK in a dose-dependent manner by phosphorylation of the TEY motif.

### Salt-induced activation of SIMK correlates with its relocation to cytoplasmic compartments in intact *M. sativa* roots

**Fig. 4.**
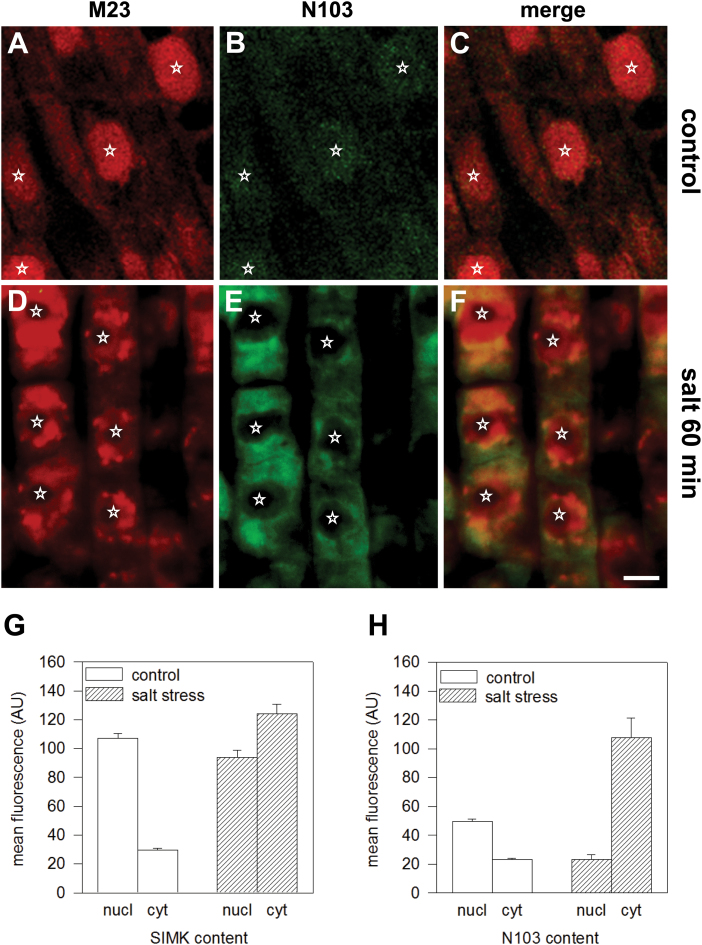
Co-localization of total and active SIMK using double immunolabelling. (A–C) Immunolabelling with protein-specific antibody M23 (A, directly labelled with Alexa Fluor 568, red) and phospho-specific antibody N103 (B, directly labelled with Alexa Fluor 488, green) in control non-dividing elongating epidermal root cells of *M. sativa*. The merged image is shown in (C). (D–F) Immunolabelling with protein-specific antibody M23 (D, directly labelled with Alexa Fluor 568, red) and phospho-specific antibody N103 (E, directly labelled with Alexa Fluor 488, green) in salt-stressed (250mM NaCl for 60min) non-dividing elongating epidermal root cells of *M. sativa*. The merged image is shown in (F). Nuclei are indicated with stars. (G, H) Microfluorometric measurements showing the change in nucleocytoplasmic redistribution of the total (G) and activated (H) pool of SIMK in control (open bars) and salt-stressed (hatched bars) *M. sativa* root cells that were simultaneously co-immunolocalized with both M23 and N103 antibodies. Mean fluorescence is shown in arbitrary units (AU, mean±standard deviation). nucl, Nuclei, cyt, cytoplasm, *n*=30. Bar, 10 μm (A–F).

Next, the effects of salt on the subcellular localization of SIMK in *M. sativa* roots were addressed. The persistent salt-induced activation of SIMK in intact roots of *M. sativa* coincided with the depletion of SIMK from nuclei and its increased association with punctate cytoplasmic structures (Fig. 2B). As revealed by immunofluorescence microscopy using the SIMK protein-specific M23 antibody, these compartments frequently coalesced into patchy structures around nuclei (Fig. 2B; see also Fig. 4D). Partial depletion of the strong nuclear SIMK signal upon salt treatment of root cells (Fig. 2B) suggested that SIMK was partially relocated from this compartment.

**Fig. 3.**
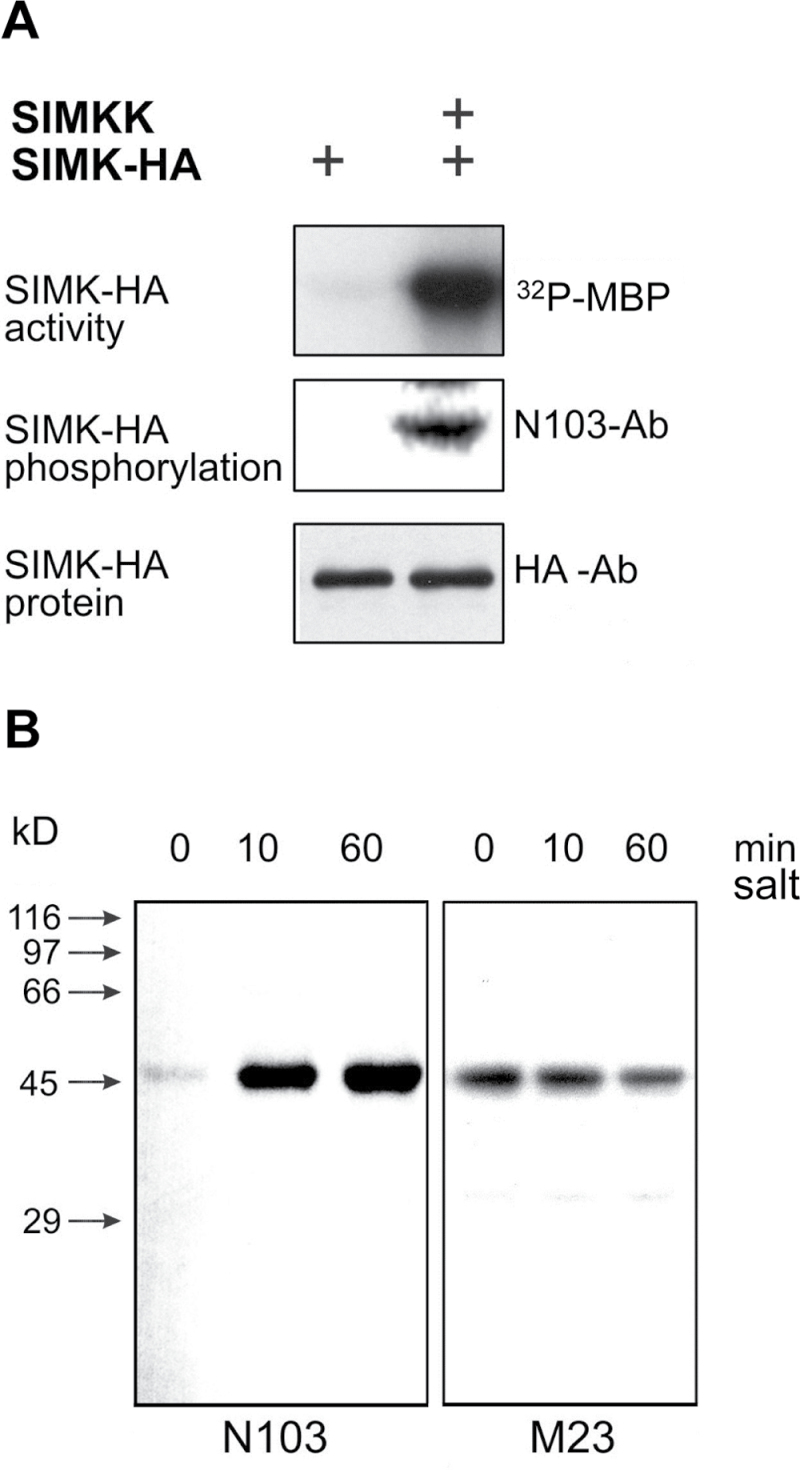
Immunokinase assays and immunoblotting with the phospho-specific and protein-specific SIMK antibodies. (A) Immunokinase assays (upper panel) and immunoblotting with phospho-specific N103 (middle panel) and HA (lower panel) antibodies in *Arabidopsis* protoplasts co-transformed with HA-tagged SIMK and its upstream activator SIMKK. Note that the N103 antibody recognizes SIMK activated by its upstream activator SIMKK. (B) Immunoblotting with the phospho-specific N103 and protein-specific SIMK antibodies in control and salt-treated *M. sativa* roots. Roots were treated with 250mM NaCl for the indicated times and crude extracts from roots were subjected to immunoblotting using N103 and M23. In control untreated roots (0min), extremely weak labelling with N103 was obtained whereas after 10 and 60min treatment with salt, N103 recognized a 46kDa band corresponding to SIMK, as revealed by the M23 antibody in the same experiment. Note that labelling with N103 got slightly stronger with increased time of root exposure to the salt stress.

Next, it was examined whether salt-induced activation of SIMK was correlated with its relocation. Therefore, the SIMK activation state within the nucleus and the cytoplasmic compartments were studied by using the phospho-specific antibody N103 (raised against the SIMK phosphopeptide CTDFMpTEpYVVTRWC; see also Šamaj *et al.*, 2002). Specificity tests revealed that the N103 antibody recognized activated SIMK in *Arabidopsis* protoplasts upon co-expression of HA-tagged SIMK and its upstream activator SIMKK (Fig. 3A). Immunoblots using crude *M. sativa* root extracts and N103 antibody showed little active SIMK in control roots (Fig. 3B, 0 min). Within 10min of salt treatment, however, N103 antibody clearly recognized a 46kDa band, which corresponded to SIMK (Fig. 3B). These data showed that salt stress in *M. sativa* roots activates SIMK through dual phosphorylation of the TEY motif.

In order to localize the pool of active SIMK, M23 (protein-specific) and N103 (activity-specific) SIMK antibodies were covalently coupled to two different fluorochromes (Alexa Fluor 568 and 488, respectively). In control *M. sativa* root cells, the co-localization experiments revealed a predominant nuclear localization of SIMK (Fig. 4A) and very low levels of dually phosphorylated SIMK (Fig. 4B, C). In contrast, after salt treatment, a significant amount of dual-phosphorylated SIMK was found in the cytoplasm and mostly in patchy structures around the nuclei (Fig. 4D–F). Although some inactive SIMK remained in the nuclei of salt-treated roots (Fig. 4D), these data suggested that salt stress-induced dual phosphorylation and activation of SIMK induced its relocation to the cytoplasm in punctate compartments.

To obtain semi-quantitative data for this process, microfluorimetric measurements were performed on the nucleocytoplasmic partitioning of phosphorylated versus non-phosphorylated SIMK in control and salt-stressed *M. sativa* root cells. The pools of inactive and active SIMK were quantified by measuring the mean fluorescence intensity of total SIMK immunolocalized with M23 antibody in comparison with dual-phosphorylated SIMK labelled with N103 antibody within the nuclear and cytoplasmic fractions of control and salt-treated cells (Fig. 4G, H). These measurements clearly demonstrated that a pool of non-phosphorylated SIMK was concentrated within the nuclei of control cells (Fig. 4G), whereas phosphorylated SIMK accumulated in the cytoplasm of cells treated with salt (Fig. 4H).

Altogether, both single and double immunolocalization studies together with quantitative and immunobiochemical analyses strongly suggested that salt-induced phosphorylation and activation of SIMK is correlated with its recruitment to cytoplasmic compartments in intact roots of *M. sativa*.

### Salt stress can trigger the association of both SIMKK and SIMK with cytoplasmic compartments

**Fig. 5.**
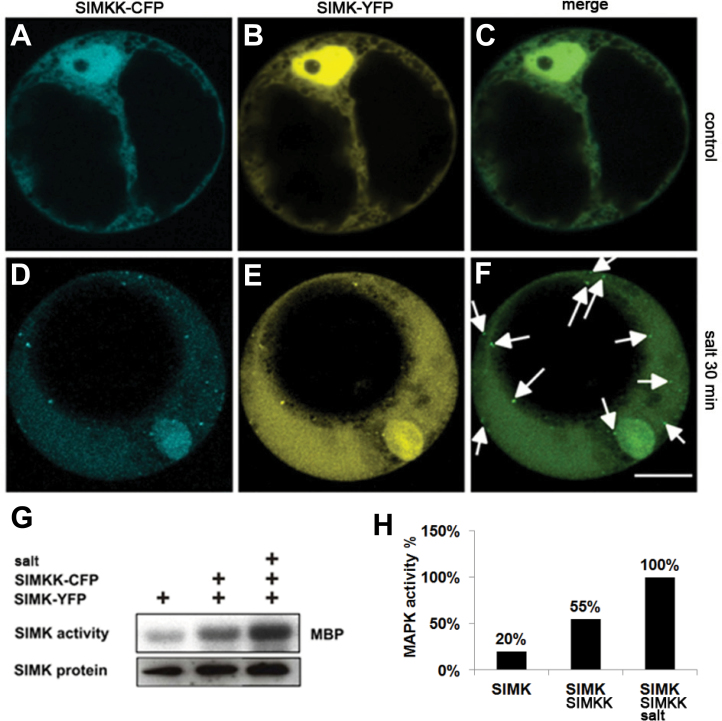
Co-localization of SIMKK and SIMK in *Arabidopsis* protoplasts. (A–C) Co-localization of CFP-tagged SIMKK (A) and YFP-tagged SIMK (B) in transiently co-transformed *Arabidopsis* protoplasts. In control protoplasts, both SIMKK (A) and SIMK (B) were predominantly nuclear proteins (merged image shown in C). (D–F) After salt treatment (250mM NaCl for 30min), both SIMKK (D) and SIMK (E) relocated to the cytoplasm where they co-localized on cytoplasmic punctate compartments (merged image shown in F, indicated by arrows). (G) Immunokinase assays and immunoblots showing SIMK activity (upper panel) and protein levels (lower panel) of SIMK in control and salt-treated (250mM NaCl for 30min) protoplasts co-transformed with SIMKK–CFP and SIMK–YFP. (H) Maximal activity of SIMK in salt-treated cells co-transformed with SIMKK. Bar, 10 μm (A–F).

Both SIMKK and SIMK were tagged with different fluorescent proteins (CFP and YFP) and co-expressed in *Arabidopsis* protoplasts in order to investigate their co-localization. Under control conditions, both CFP-tagged SIMKK and YFP-tagged SIMK preferentially localized to the nuclei, but they were also dispersed throughout the cytoplasm (Fig. 5A–C). In contrast, both SIMKK–CFP and SIMK–YFP relocated to cytoplasmic punctate compartments where they co-localized after 30min of salt treatment (Fig. 5D–F). Immunoprecipitation with GFP- and SIMK-specific antibodies and subsequent immunokinase assays revealed that SIMKK–CFP was functional, being able to phosphorylate SIMK–YFP (Fig. 5G, H). As SIMK protein levels remained constant in these experiments, SIMK–YFP appeared to be post-translationally activated by SIMKK–CFP (Fig. 5G). These data suggested that salt treatment triggered SIMKK–CFP mediated activation of SIMK–YFP, which was associated with concomitant enrichment and co-localization of both kinases in cytoplasmic compartments. Relocation and co-localization of both SIMK and SIMKK to cytoplasmic compartments suggested that both kinases were actively targeted to these structures in a coordinated manner.

### Constitutive import of SIMKK into the nucleus under control and salt-stress conditions

To investigate the mechanism of nuclear import of SIMKK in living cells, FRAP was used in *Arabidopsis* protoplasts transformed with SIMKK–YFP. As the YFP-tagged SIMKK fusion protein is much larger (>70kDa) than the exclusion limit of nuclear pores (maximum 45–50kDa), it requires an active nuclear import mechanism. During laser bleaching of the nuclear compartment, the whole nuclear area was first depleted and subsequently replenished quickly, indicating that YFP-tagged SIMKK was mobile. During the recovery, we always observed a simultaneous overall decrease in cytoplasmic fluorescence with increased time (Supplementary Fig. S1A available at *JXB* online). These data suggested that cytoplasmic SIMKK–YFP steadily enters the nuclei. The FRAP rate of SIMKK–YFP in bleached nuclei was similar between control and salt-stressed cells, although the slope of the curves reflected some differences (Supplementary Fig. S1B, C). After photobleaching of nuclei, nuclear SIMKK–YFP fluorescence recovered at similar rates in control and salt-stressed *Arabidopsis* protoplasts, with half-life times between 31.5 and 32.5 s (Supplementary Fig. S1D). The immobile protein fractions, however, showed a significant reduction after salt stress (Supplementary Fig. S1E). These results suggested that, although the mobility of SIMKK–YFP protein is not considerably affected by salt, the mobile pool of SIMKK–YFP in the nucleus is altered, probably as a consequence of salt-induced SIMKK–YFP activation.

### Salt-induced accumulation of YFP-tagged SIMKK into cytoplasmic compartments in *Arabidopsis* plants

**Fig. 6.**
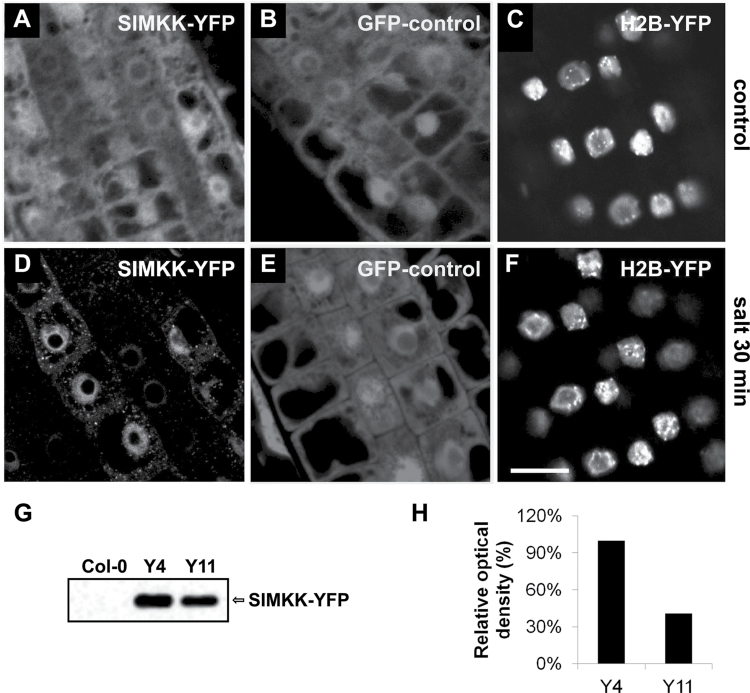
Localization of SIMKK in non-dividing epidermal cells in the elongating zone of the root tip of the stable transformed *Arabidopsis* line Y11. (A) YFP-tagged SIMKK in root epidermal cells under control conditions. (B) Localization of GFP in line G5 expressing GFP alone under control conditions. (C) Localization of nuclear marker H2B–YFP under control conditions. (D) YFP-tagged SIMKK in root epidermal cells upon salt stress (250mM NaCl for 30min). (E) Localization of GFP in line G5 expressing GFP alone upon salt stress (250mM NaCl for 30min). (F) Localization of nuclear marker H2B–YFP upon salt stress (250mM NaCl for 30min). (G) Immunoblot analysis of the recombinant SIMKK–YFP protein expression in roots of two *Arabidopsis* lines (Y4 and Y11) overexpressing SIMKK–YFP and wild type (Col-0) using anti-GFP antibody. A band corresponding to the recombinant protein with molecular weight of 70kDa is shown. (H) Graph showing the relative optical density of bands in (G) corresponding to SIMKK–YFP. Bar, 10 μm (A–F).

In order to extend our results with transiently transformed protoplasts and *M. sativa* root cells, we generated 19 independent *Arabidopsis* lines stably expressing CFP- or YFP-tagged SIMKK under the 35S CaMV promoter. Fluorescently (CFP or YFP) tagged SIMKK was predominantly nuclear under control conditions in epidermal cells of *Arabidopsis* root tip (Fig. 6A), but it was enriched in cytoplasmic punctate compartments following salt treatment (Fig. 6D). On the other hand, the distribution of fluorescence was identical in control plants transformed with GFP, irrespectively of salt treatment, showing GFP accumulation within the nuclei but no association with cytoplasmic compartments after salt treatment (Fig. 6B, E). As another control, nuclear protein histone 2B (H2B) tagged with YFP was used to confirm the integrity of nuclei under the plasmolytic conditions caused by 250mM NaCl. Similarly to GFP alone, H2B–YFP was also not associated with cytoplasmic compartments upon salt stress but rather showed exclusive and constitutive nuclear localization (Fig. 6C, F).

The expression level of the SIMKK–YFP in independently transformed *Arabidopsis* lines fluctuated according to the roughly estimated fluorescence intensity. Therefore, we characterized the level of SIMKK–YFP expression in two selected lines (Y4 and Y11) by immunoblotting using anti-GFP antibody recognizing the YFP tag of SIMKK. The expression level of SIMKK–YFP in line Y4 was 2.5-fold higher than in line Y11 (Fig. 6G, H). As the levels of constitutively overexpressed SIMKK may considerably influence properties of transformed plants, these two lines with altered levels of SIMKK–YFP overexpression were further studied by phenotypic and biochemical analyses for their sensitivity to salt stress.

### Overexpression of SIMKK in *Arabidopsis* causes enhanced MPK6 and MPK3 activation and altered sensitivity to salt stress

**Fig. 7.**
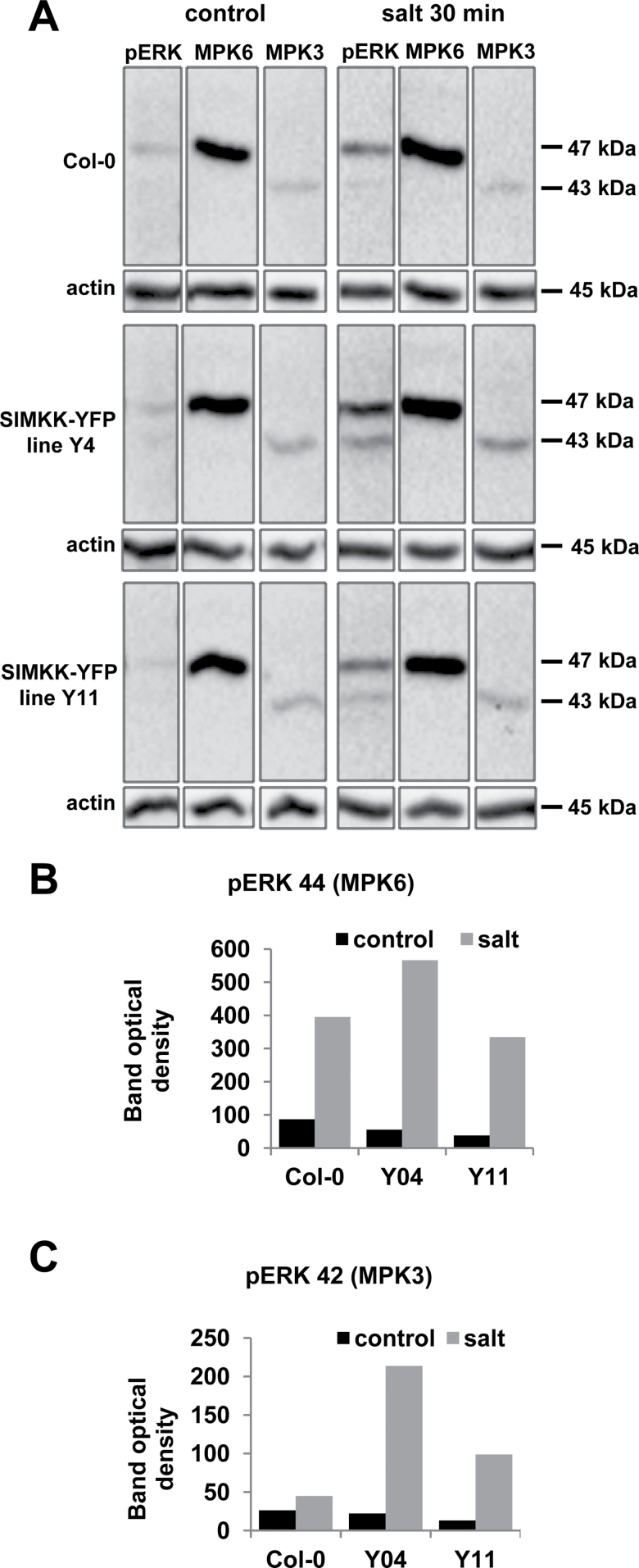
Salt stress-induced MPK6 and MPK3 activation in stably transformed *Arabidopsis* lines Y4 and Y11 overexpressing SIMKK–YFP. Seedlings (16 d old) of line Y4, line Y11, and Col-0 (control) grown on solidified culture medium were treated with 250mM NaCl for 30min. (A) Root protein extracts separated by SDS-PAGE were probed with affinity-purified antibodies against mammalian ERK1/2 (phospho-p44/42, pERK), *Arabidopsis* MPK6, and *Arabidopsis* MPK3. Molecular mass is indicated. Staining with anti-actin antibody was used as a loading control. (B, C) Graphs showing quantitative analysis of the relative optical densities of pERK bands corresponding to activated MPK6 (B) and activated MPK3 (C) in control and salt-treated samples.

Stably transformed *Arabidopsis* lines overexpressing SIMKK–YFP and non-transformed Col-0 seedlings, untreated or treated with 250mM NaCl for 30min, were analysed and compared for MPK6 and MPK3 expression and activation. Protein extracts separated by SDS-PAGE were probed with affinity-purified antibodies against the dual-phosphorylated pTEpY motif of mammalian ERK1/2 (phospho-p44/42), and with affinity-purified antibodies against *A. thaliana* MPK6 and MPK3. The protein separation setup allowed unambiguous discrimination between MPK6 and MPK3 in identical SDS-PAGE-separated samples using commercially available, affinity-purified MPK6 and MPK3 antibodies (Fig. 7). The activation of MPK6 and MPK3 was studied using the pTEpY-specific phospho-p44/42 antibody. Its application identified a band persisting at ~46kDa in Col-0 plants in control conditions, which, by size comparison, clearly corresponded to MPK6 (Fig. 7). This band was evident in all non-induced samples, including the stably transformed lines Y11 and Y4 (Fig. 7). However, the second band at ~43kDa, corresponding to MPK3, was weakly detectable in Y4 but absent in Col-0 and Y11 plants (Fig. 7). A 30min salt treatment induced phosphorylation of both MPK6 and MPK3 in Col-0 and Y11 plants but considerably stronger activation in the Y4 line (Fig. 7). These data suggested that SIMKK–YFP overexpression in *Arabidopsis* causes the upregulation of both MPK6 and MPK3 kinase activities (Fig. 7) in a quantitative manner that correlates with the level of overexpression of the MAPKK (Fig. 6G, H).

**Fig. 8.**
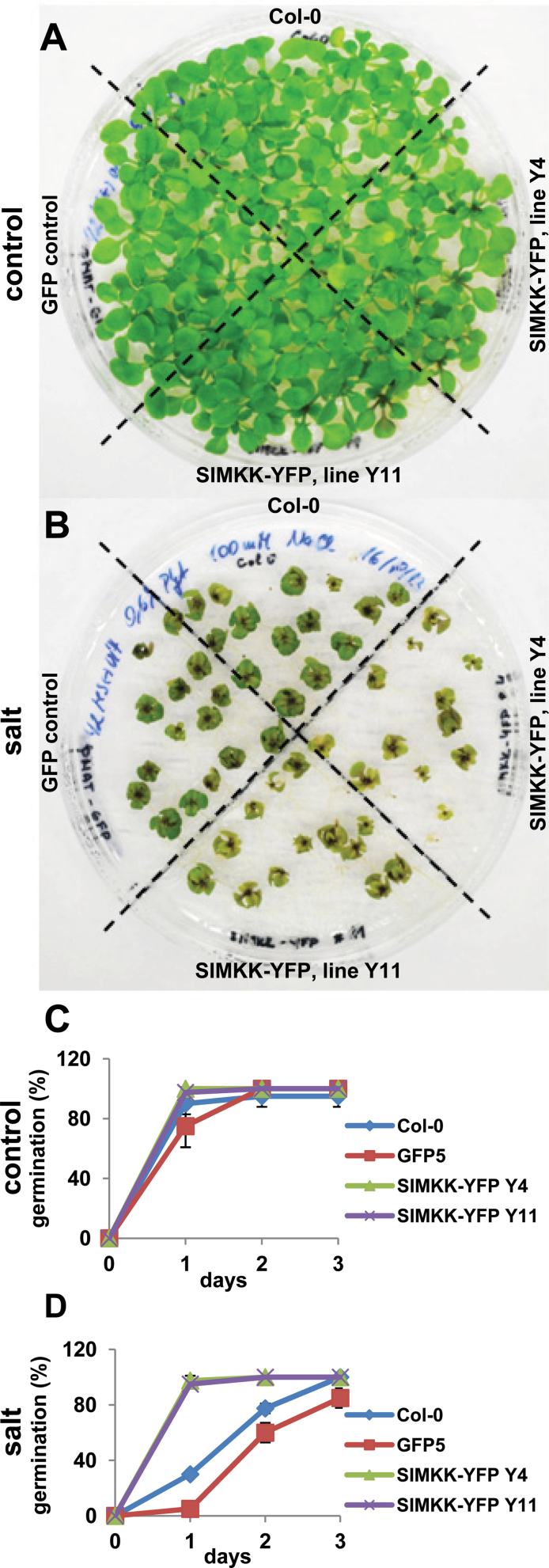
Salt sensitivity test of *Arabidopsis* plants stably transformed with YFP-tagged SIMKK. (A, B) Plates containing control plants of wild-type Col-0, control plants stably transformed with GFP alone (line G5), and transformed plants expressing YFP-tagged SIMKK (lines Y4 and Y11). Five-day-old seedlings germinating on control medium were transferred to control medium (A), and to medium containing 100mM NaCl (B). Plants were photographed 14 d after the transfer. (C, D) Evaluation of the germination rate of wild-type Col-0, line G5, and YFP-tagged SIMKK lines Y4 and Y11 in control medium (C) and medium containing 100mM NaCl (D).

Importantly, the SIMKK–YFP overexpressor lines Y4 and Y11 were clearly more sensitive to salt when 5-d-old plants were transferred for long-term growth on 100mM NaCl for 14 d (Fig. 8A, B). Comparison of Y4 and Y11 lines revealed that line Y4 was more sensitive to salt because more plants of this line exhibited dwarfism or completely inhibited growth (Fig. 8B). These data suggested that constitutive overexpression of SIMKK–YFP in growing *Arabidopsis* plants compromises their ability to withstand moderate salt stress, and it was, at least partially, related to the level of SIMKK–YFP overexpression in the Y4 and Y11 lines. These *Arabidopsis* lines, however, were salt insensitive during germination (Fig. 8C, D; Supplementary Figs S2 and S3). The two SIMKK–YFP overexpressing lines Y4 and Y11, as well as wild-type Col-0 and a GFP overexpressing line germinated effectively within 24h in control medium (Fig. 8C; Supplementary Fig. S3A available at *JXB* online). On d 2 after germination, cotyledons and elongating roots with protruding root hairs were clearly visible in all lines (Supplementary Fig. S3B). Although the germination was not absolutely synchronous (reaching 80–90% in control lines and 100% in SIMKK–YFP overexpressing lines within the first 24h), it was completed for all lines by d 2 (Fig. 8C; Supplementary Fig. S2A available at *JXB* online). Germination of wild-type Col-0 and a GFP overexpressing line in the presence of 100mM NaCl was considerably delayed, whilst SIMKK–YFP overexpressing lines germinated effectively without any reduction (Fig. 8D; Supplementary Fig. S3A). Accordingly, post-germination growth of SIMKK–YFP overexpressing lines on d 2 was not much influenced by salt in the medium, showing minimal differences to SIMKK–YFP overexpressing plants growing in control medium (Supplementary Fig. S3B). Long-term growth under these conditions, however, revealed salt susceptibility of SIMKK–YFP overexpressing lines, as they were not able to survive moderate salt stress after 23 d (Supplementary Fig. S2C). These data clearly indicated a large insensitivity of SIMKK–YFP overexpressing lines to moderate salt stress at the stage of germination but enhanced salt sensitivity of developing plants after their exposure to NaCl. Although the constitutive overexpression of SIMKK–YFP in *Arabidopsis* seems to be responsible for this developmentally regulated reaction to salt stress, it must be tightly linked to other stress-related components of the endogenous signalling pathway that are regulated in the same way.

### Proteomic analysis of SIMKK–YFP overexpressor plants reveals a lower abundance of stress-related proteins

Proteomic analysis was performed to better characterize SIMKK–YFP overexpressor plants at the molecular level. Protein identification details from each replicate, as well as quantification of significantly differentially regulated proteins, are shown in Supplementary Tables S1 and S2, respectively, available at *JXB* online. On average, this one-dimensional LC/MS^E^ proteomic analysis resulted in the identification of 237 proteins in SIMKK–YFP transgenic plants and 256 proteins in the wild-type plants (Supplementary Table S1). Of these, 22 proteins were detected as significantly differentially abundant between SIMKK–YFP transgenic plants and wild-type Col-0 plants (Table 1). Ten of these proteins are involved in stress responses, representing the most abundant functional class of differentially regulated proteins (Table 1). Notably, proteins involved in salt-induced oxidative stress (e.g. in hydrogen peroxide detoxification) such as catalase, peroxiredoxin, and glutathione *S*-transferase, as well as nucleoside diphosphate kinase 1, a signalling protein involved in reactive oxygen species signalling and interacting with catalase (Fukamatsu *et al.*, 2003), were all less abundant in transgenic SIMKK–YFP plants.

**Table 1.** Overview of differentially abundant root proteins in Arabidopsis transgenic plants overexpressing SIMKK–YFP (line 11) compared with the wild-type Col-0 plants

Accession	Description	mW (Da)	pI (pH)	Peptides	Coverage (%)	Average amount in Col-0 (fmol)	Average amount in SIMKK–YFP (fmol)	fold change	*P* value
**Stress-related**									
B9DFK6	Catalase 2	54990	6.35	3	8.23	243.00	71.00	0.29	0.010
O80852	Glutathione *S*-transferase F9	24130	6.18	9	28.84	2113.75	1028.75	0.49	0.020
Q9SYT0	Annexin D1	36181	5.05	6	22.08	890.66	446.66	0.50	0.050
F4ID64	Peroxiredoxin 2B	13037	5.41	2	42.98	536.41	398.95	0.74	0.045
O04311	Jacalin-like lectin domain- containing protein	32002	4.90	11	36.33	731.50	1171.50	1.60	0.050
O80912	Peroxidase 23	38076	7.95	9	14.33	498.50	823.33	1.65	0.040
Q96511	Peroxidase 69	35656	9.62	3	17.52	384.33	666.00	1.73	0.028
Q9SUR0	Putative major latex protein	17506	5.90	17	43.05	2651.50	5031.75	1.90	0.001
P94014	Germin-like protein subfamily 2 member 1	22853	8.85	3	34.24	199.00	530.33	2.66	0.050
Q9FF53	Probable aquaporin PIP2	30931	8.09	3	21.65	140.50	464.00	3.30	0.050
**Cytoskeletal proteins**									
Q42449	Profilin 1	14257	4.50	4	22.90	625.33	381.33	0.61	0.004
**Metabolic proteins**									
Q0WP70	5-Methyltetrahydropteroyltriglutamate homocysteine *S*-methyltransferase- like protein	65692	6.22	5	9.00	63.00	29.66	0.47	0.010
Q9LYG3	NADP dependent malic enzyme 2	64372	5.97	9	32.65	1242.75	606.25	0.49	0.008
Q8H135	ATP synthase subunit β	48227	5.28	21	46.19	1918.66	1069.00	0.56	0.001
Q9LZG0	Adenosine kinase 2	37821	4.97	6	25.51	319.33	194.00	0.61	0.003
Q9SR37	β-Glucosidase 23	59682	6.46	36	36.45	6292.50	9042.00	1.44	0.030
Q56WD9	3-Ketoacyl CoA thiolase 2 peroxisomal	48548	8.35	4	16.88	303.25	476.75	1.57	0.005
**Proteolysis and protein processing**									
P30184	Leucine aminopeptidase 1	54475	5.55	6	22.69	296.75	489.75	1.65	0.002
Q9LMU2	Kunitz type trypsin and protease inhibitor domain-containing protein	22067	8.97	4	18.36	1004.66	541.33	0.54	0.001
**Lipid binding**									
Q56WK6	Patellin 1	64007	4.62	10	18.15	302.97	524.78	1.73	0.050
**Protein folding**									
F4K007	Luminal-binding protein 2	67358	4.99	15	23.00	103.00	522.00	5.07	0.050
**Signalling**									
P39207	Nucleoside diphosphate kinase 1	16489	6.36	4	22.82	528.36	273.75	0.52	0.002

Two peroxidases, namely peroxidase 69 and peroxidase 23, belonging to the class III of peroxidase superfamily, were slightly upregulated in the transgenic plants (Table 1). Aquaporin PIP2, which is involved in water transport across the plasma membrane, was more abundant in transgenic SIMKK–YFP plants (Table 1), indicating the accelerated water conductance followed by increased loss of water (Katsuhara *et al.*, 2003). Germin-like protein subfamily 2 member 1, upregulated 2.6-fold in SIMKK–YFP plants (Table 1), was shown to be a plasmodesmata-localized protein, which facilitates the plasmodesmata permeability when overexpressed in *Arabidopsis* (Ham *et al.*, 2012). Furthermore, the actin-binding protein profilin 1, which promotes actin polymerization (Staiger *et al.*, 2010) was also downregulated in these transgenic plants (Table 1). Upregulation of luminal-binding protein 2 suggested altered protein folding in the SIMKK–YFP plants.

## Discussion

MAPK signalling cascades regulate cellular processes at different subcellular compartments. A proper understanding of the spatiotemporal organization of individual MAPK modules requires studying the dynamic localization and mode of activation/inactivation of MAPKs during plant development and stress responses (Šamajová *et al.*, 2013*b*). The data presented in this work provide compelling evidence of a coordinated and activity-dependent relocation of SIMKK and SIMK during salinity stress in plant cells. In a complementary transgenic approach in *Arabidopsis*, SIMKK–YFP overexpression resulted in enhanced MPK3 and MPK6 kinase activation and conferred altered sensitivity to salt stress. Considering that SIMKK-overexpressing plants have changed amounts of some proteins functioning in salt and oxidative stress responses, these data suggest that SIMKK may play a role as possible negative regulator of salt stress tolerance.

Phospho-specific anti-pTEpY antibodies allow the identification of the activated MAPKs at the cellular level. With such an antibody, it was shown previously that activated SIMK is recruited to tips of growing root hairs (Šamaj *et al.*, 2002). Here, we aimed to study the activation and localization of SIMK upon salt stress in intact roots, representing a plant organ that typically has to cope with increased soil salinity in nature. We showed that SIMK is persistently activated in intact *Medicago* roots and enriched in cytoplasmic compartments following salt treatment. Our data are supported by several independent lines of evidence: (i) immunokinase assays and immunoblotting with phospho-specific antibodies, both showing persistent activation of SIMK in intact roots upon salt stress; (ii) immunolocalization with protein- and phospho-specific antibodies, both showing relocation of SIMK to cytoplasmic compartments in intact roots; (iii) *in vivo* relocation of SIMK to cytoplasmic compartments in non-dividing protoplasts; and (iv) similar relocations of SIMKK (involved in salt stress activation of SIMK) into cytoplasmic compartments in root cells and non-dividing protoplasts.

The immunolocalization results with phospho-specific SIMK antibody demonstrated the requirement of SIMK activation for its relocation and helped to explain the coordinated accumulation of SIMKK and SIMK into cytoplasmic compartments during salt stress. It is possible that SIMKK functions as a scaffold protein for SIMK or, alternatively, other unknown scaffold proteins that bind both activated SIMKK and SIMK could be involved in their coordinated relocation to cytoplasmic compartments upon salt stress. It is interesting to note that in this context activated kinase modules have been localized to clathrin-coated vesicles and signalling endosomes in animals (Howe *et al.*, 2001; Sorkin and von Zastrow, 2002), but so far nothing is known about similar processes in plants. Our recent data showed the co-localization of *Arabidopsis* MPK6 and clathrin at the plasma membrane and at TGN vesicles (Müller *et al.*, 2010). These findings are consistent with previous reports suggesting cytoplasmic functions for plant MAPKs and MAPKKKs in specific situations such as cell division (Calderini *et al.*, 1998; Bögre *et al.*, 1999; Nishihama *et al.*, 2001) and root hair development (Šamaj *et al.*, 2002), both representing processes associated with vigorous vesicular trafficking.

The dynamic relocalization of the MAPK and MAPKK components from the nucleus to the cytoplasm seems to challenge the traditional view of MAPK translocation from the cytoplasm to the nucleus upon its activation. SIMK and SIMKK relocation into the cytoplasmic compartments upon salt treatment could represent an intermediate stage within the activation-dependent relocation to certain membrane targets that are supposed to be regulated upon salt stress. Supporting evidence that the process of activated MAPK relocation to membrane targets may be related to plant adaptation strategies to salt stress comes from studies of the salt overly sensitive (SOS) pathway. Within this pathway, a complex of SOS2, a Ser/Thr protein kinase (Liu *et al.*, 2000), with SOS3, a myristoylated calcium-binding protein (Ishitani *et al.*, 2000), phosphorylates and activates the transport activity of SOS1, a plasma membrane Na^+^/H^+^ antiporter (Shi *et al.*, 2000; Qiu *et al.*, 2002). MPK6 phosphorylates and activates SOS1, whilst the MAPK module encompassing MPK6 is located at the plasma membrane (Yu *et al.*, 2010; Kim *et al.*, 2012).

Salt stress triggers different MAPK signalling pathways in *Arabidopsis* resulting in positive or negative outcomes in terms of plant tolerance. It is well documented that both MPK6 and MPK3 are activated by NaCl stress, and their activation seems to be required for salt tolerance in *A. thaliana* (Ichimura *et al.*, 2000; Droillard *et al.*, 2002; Teige *et al.*, 2004; Yu *et al.*, 2010). High concentrations of salt activate MKKK20 in *Arabidopsis*, which is upstream of MPK6 (Kim *et al.*, 2012), but in the MEKK1-dependent signalling pathway involving MPK4, *mekk1* mutants exhibited improved growth under high salinity (Su *et al.*, 2007). These results suggest that, in contrast to MKKK20, MEKK1 may negatively regulate the salt response in *Arabidopsis*. MKK2 is another major activator of MPK4 and MPK6 in cold and salinity conditions, as *mkk2* null mutants show salt hypersensitivity (Teige *et al.*, 2004). On the other hand, loss of MKK9 activity as an activator of MPK6 reduced salt sensitivity, indicating its negative impact in salinity tolerance (Xu *et al.*, 2008). Similar results have been shown in *mkk1* knockout plants, which were able to tolerate elevated salt concentrations in both germination and post-germination seedling development, indicating that MKK1 is probably a negative regulator of salt stress (Conroy *et al.*, 2013).

Generation of transgenic *Arabidopsis* lines overexpressing orthologous members of MAPK signalling pathways from other species facilitates their functional characterization (Ren *et al.*, 2002; Han *et al.*, 2010). In this study, constitutive overexpression of YFP-tagged SIMKK in *Arabidopsis* resulted in lower abundance of some salt-stress-related proteins in roots and enhanced salt stress sensitivity at the stage of seedling growth. It is well known that salt stress induces reactive oxygen species production and antioxidant defence in plants (Munns and Tester, 2008; Miller *et al.*, 2010), whilst increased antioxidant defence is linked to higher tolerance against salinity (Badawi *et al.*, 2004; Ashraf, 2009). *Arabidopsis* plants overexpressing SIMKK–YFP exhibited constitutively decreased abundance of proteins such as catalase, glutathione *S*-transferase and peroxiredoxin, all involved in antioxidant defence. Other enzymes putatively involved in hydrogen peroxide decomposition, class III peroxidases (peroxidase 23 and 69), were slightly upregulated in the transgenic plants. These enzymes have, except for hydrogen peroxide-decomposing activity, multiple functions in cells including cell-wall metabolism (Passardi *et al.*, 2004), and they can generate highly reactive oxygen species (Liszkay *et al.*, 2003). This suggests that the contribution of peroxidases to hydrogen peroxide removal in SIMKK–YFP plants is not unambiguous and they may exert functions that are not connected to salt-induced oxidative burst.

In addition, nucleoside diphosphate kinase 1 was downregulated in these transgenic plants. Interestingly, nucleoside diphosphate kinases interact with catalase and are involved in salt-stress tolerance (Fukamatsu *et al.*, 2003; Verslues *et al.*, 2007). Thus, SIMKK overexpression might negatively affect nucleoside diphosphate kinase 1-mediated signalling towards catalase, leading to increased sensitivity to salt in SIMKK–YFP plants. Other molecular mechanisms determining the higher susceptibility of SIMKK–YFP-overexpressing plants to salt stress may be suggested by the increased constitutive abundance of the aquaporin PIP2, as shown by the proteomic analysis. The accelerated water conductance driven by the higher abundance of aquaporins (Javot *et al.*, 2003) together with restricted capacity of plants for water uptake under salt stress (Romero-Aranda *et al.*, 2001) may result in the limited growth and development of SIMKK–YFP transgenic plants. This mechanism was also suggested for rice plants overexpressing barley HvPiP2.1, which showed higher sensitivity to salt stress (Katsuhara *et al.*, 2003). Reduction of the hydraulic conductivity in *Arabidopsis* root cells upon NaCl treatment was caused not only by an overall decrease in the abundance of aquaporins but also by their relocation from the plasma membrane (Boursiac *et al.*, 2005). In addition to this stimulus-dependent relocation of aquaporins, stress-induced changes in their phosphorylation status have been described. Phosphorylation of aquaporins is required for regulation of their gating (Tornroth-Horsefield *et al.*, 2006). However, in response to salinity stress, specific changes in the C-terminal phosphorylation of AtPIP2;1 were characterized, and these were related to relocation of AtPIP2;1 from the plasma membrane to intracellular compartments (Prak *et al.*, 2008). Abundance of aquaporins, their phosphorylation status, and their subcellular localization in particular developmental stages may act as one of the key determinants of altered salt susceptibility of SIMKK–YFP-overexpressing plants. Obviously, the relevance of aquaporins as potential substrates for MAPK-mediated phosphorylation should be addressed in future studies, as it might be an important aspect of the complex reactions of plants to abiotic stresses. Germin-like protein subfamily 2 member 1 was defined as a protein localized to the plasmodesmata. This protein contributes to the increased plasmodesmata conductance and possibly enhances the symplastic intercellular water transport. The overexpression of SIMKK–YFP in *Arabidopsis* also led to substantial upregulation of molecular chaperone luminal-binding protein 2, perhaps indicating the demand for accelerated protein folding in these plants. The biological relevance of this phenomenon remains to be substantiated experimentally.

In conclusion, this study has revealed dynamic aspects of MAPK signalling components of intact plants challenged by salt stress and has provided compelling evidence that coordinated and activity-dependent relocations of SIMKK and SIMK are tightly linked with salt stress in plant cells. Overexpression of SIMKK in *Arabidopsis* plants leads to higher activation of endogenous MPK6 and MPK3 following short-term salt treatment, whilst it results in enhanced sensitivity of these plants to long-term salinity stress. Overall, our study revealed that MAPK signalling is a highly coordinated process in both space and time that is still poorly understood and needs to be investigated in more depth. In addition, our work also indicates that a predictive biological engineering of signalling pathways will require a much better understanding of the full set of protein partners that make up the signalling pathways and interact with each other in a given process.

## Supplementary data

Supplementary data are available at *JXB* online.

Supplementary Fig. S1. Fluorescence recovery after photobleaching (FRAP) analysis of YFP-tagged SIMKK in transiently transformed *Arabidopsis* protoplasts.

Supplementary Fig. S2. Overview of germination test of *Arabidopsis* plants stably transformed with YFP-tagged SIMKK under salt stress.

Supplementary Fig. S3. Details of germination test of *Arabidopsis* plants stably transformed with YFP-tagged SIMKK under salt stress.

Supplementary Video S1. SIMKK–YFP in *Medicago sativa*, salt stress. This movie shows transiently transformed *M. sativa* root epidermal cell. The SIMKK–YFP is localized to the nucleus and cytoplasmic vesicles exhibiting fast movements. Salt treatment is enhancing relocation of SIMKK–YFP to these cytoplasmic vesicles. The time lapse covers a period of 15 s (accelerated 2.4 times; avi; 26.1 MB).

Supplementary Table S1. Details of protein identification and quantification for biological replicates pertinent for proteomic analysis of roots of SIMKK–YFP transgenic plants and wild-type plants.

Supplementary Table S2. The quantification of significantly differentially abundant root proteins between SIMKK–YFP transgenic plants and wild-type plants.

Supplementary Data

## Abbreviations:

CaMV: cauliflower mosaic virus
CFP: cyan fluorescent protein
FRAP: fluorescence recovery after photobleaching
GFP: green fluorescent protein
HA: haemagglutinin
LC: liquid chromatography
MAPK: mitogen-activated protein kinase
MS: mass spectrometry
Q-TOF: quadrupole time-of-flight
SIMK: stress-induced MAPK
UPLC: ultraperformance liquid chromatography
YFP: yellow fluorescent protein.

## References

[CIT0001] Ashraf M 2009 Biotechnological approach of improving plant salt tolerance using antioxidants as markers. Biotechnology Advances 27, 84–93 18950697 10.1016/j.biotechadv.2008.09.003

[CIT0002] Badawi GH Yamauchi Y Shimada E Sasaki R Kawano N Tanaka K Tanaka K 2004 Enhanced tolerance to salt stress and water deficit by overexpressing superoxide dismutase in tobacco (*Nicotiana tabacum*) chloroplasts. Plant Science 166, 919–928

[CIT0003] Beck M Komis G Müller J Menzel D Šamaj J 2010 *Arabidopsis* homologs of nucleus- and phragmoplast-localized kinase 2 and 3 and mitogen-activated protein kinase 4 are essential for microtubule organization. Plant Cell 22, 755–771 20215588 10.1105/tpc.109.071746 PMC2861451

[CIT0004] Beck M Komis G Ziemann A Menzel D Šamaj J 2011 Mitogen-activated protein kinase 4 is involved in the regulation of mitotic and cytokinetic microtubule transitions in *Arabidopsis thaliana* . New Phytologist 189, 1069–1083 21155826 10.1111/j.1469-8137.2010.03565.x

[CIT0005] Boursiac Y Chen S Luu D-T Sorieul M van den Dries N Maurel C 2005 Early effects of salinity on water transport in *Arabidopsis* roots. Molecular and cellular features of aquaporin expression. Plant Physiology 139, 790–805 16183846 10.1104/pp.105.065029 PMC1255996

[CIT0006] Bögre L Calderini O Binarová P 1999 A MAP kinase is activated late in plant mitosis and becomes localized to the plane of cell division. Plant Cell 11, 101–114 9878635 PMC144092

[CIT0007] Calderini O Bögre L Vicente O Binarová P Heberle-Bors E Wilson C 1998 A cell cycle regulated MAP kinase with a possible role in cytokinesis in tobacco cells. Journal of Cell Science 111, 3091–3100 9739082 10.1242/jcs.111.20.3091

[CIT0008] Calderini O Glab N Bergounioux C Heberle-Bors E Wilson C 2001 A novel tobacco mitogen-activated protein (MAP) kinase kinase, NtMEK1, activates the cell cycle-regulated p43Ntf6 MAP kinase. Journal of Biological Chemistry 276, 18139–18145 11278711 10.1074/jbc.M010621200

[CIT0009] Cardinale F Jonak C Ligterink W Niehaus K Boller T Hirt H 2000 Differential activation of four specific MAPK pathways by distinct elicitors. Journal of Biological Chemistry 275, 36734–36740 10973984 10.1074/jbc.M007418200

[CIT0010] Cardinale F Meskiene I Ouaked F Hirt H 2002 Convergence and divergence of stress-induced mitogen-activated protein kinase signaling pathways at the level of two distinct mitogen-activated protein kinase kinases. Plant Cell 14, 703–711 11910015 PMC150590

[CIT0011] Cargnello M Roux PP 2011 Activation and function of the MAPKs and their substrates, the MAPK-activated protein kinases. Microbiology and Molecular Biology Reviews 75, 50–83 21372320 10.1128/MMBR.00031-10 PMC3063353

[CIT0012] Colcombet J Hirt H 2008 Arabidopsis MAPKs: a complex signalling network involved in multiple biological processes. Biochemical Journal 413, 217–226 18570633 10.1042/BJ20080625

[CIT0013] Conroy C Ching J Gao Y Wang X Rampitsch C Xing T 2013 Knockout of AtMKK1 enhances salt tolerance and modifies metabolic activities in *Arabidopsis* . Plant Signaling and Behavior 8, e24206 23511202 10.4161/psb.24206 PMC3907437

[CIT0014] Coronado MJ González-Melendi P Seguí JM Ramírez C Bárány I Testillano PS Risueño MC 2002 MAPKs entry into the nucleus at specific interchromatin domains in plant differentiation and proliferation processes. Journal of Structural Biology 140, 200–213 12490168 10.1016/s1047-8477(02)00542-7

[CIT0015] Clough SJ Bent AF 1998 Floral dip: A simplified method for Agrobacterium-mediated transformation of *Arabidopsis thaliana* . The Plant Journal 16, 735–743 10069079 10.1046/j.1365-313x.1998.00343.x

[CIT0016] Dóczi R Okrész L Romero AE Paccanaro A Bögre L 2012 Exploring the evolutionary path of plant MAPK networks. Trends in Plant Science 17, 518–525 22682803 10.1016/j.tplants.2012.05.009

[CIT0017] Droillard MJ Boudsocq M Barbier-Brygoo H Lauriére C 2002 Different protein kinase families are activated by osmotic stresses in *Arabidopsis thaliana* cell suspensions. Involvement of the MAP kinases AtMPK3 and AtMPK6. FEBS Letters 527, 43–50 12220631 10.1016/s0014-5793(02)03162-9

[CIT0018] Fukamatsu Y Yabe N Hasunuma K 2003 Arabidopsis NDK1 is a component of ROS signaling by interacting with three catalases. Plant Cell Physiology 44, 982–989 14581623 10.1093/pcp/pcg140

[CIT0019] Ham BK Li G Kang BH Zeng F Lucas WJ 2012 Overexpression of Arabidopsis plasmodesmata germin-like proteins disrupts root growth and development. Plant Cell 24, 3630–3648 22960910 10.1105/tpc.112.101063 PMC3480292

[CIT0020] Han L Li GJ Yang KY Mao G Wang R Liu Y Zhang S 2010 Mitogen-activated protein kinase 3 and 6 regulate *Botrytis cinerea*-induced ethylene production in Arabidopsis. The Plant Journal 64, 114–127 20659280 10.1111/j.1365-313X.2010.04318.x

[CIT0021] Howe CL Valletta JS Rusnak AS Mobley WC 2001 NGF signalling from clathrin-coated vesicles: evidence that signalling endosomes serve as a platform for the Ras-MAPK pathway. Neuron 32, 801–814 11738027 10.1016/s0896-6273(01)00526-8

[CIT0022] Hurkman WJ Tanaka CK 1986 Solubilization of plant membrane proteins for analysis by two-dimensional gel electrophoresis. Plant Physiology 81, 802–806 16664906 10.1104/pp.81.3.802 PMC1075430

[CIT0023] Ichimura K Mizoguchi T Yoshida R Yuasa T Shinozaki K 2000 Various abiotic stresses rapidly activate Arabidopsis MAP kinases ATMPK4 and ATMPK6. The Plant Journal 24, 655–665 11123804 10.1046/j.1365-313x.2000.00913.x

[CIT0024] Ishitani M Liu J Halfter U Kim CS Shi W Zhu JK 2000 SOS3 function in plant salt tolerance requires *N*-myristoylation and calcium-binding. Plant Cell 12, 1667–1677 11006339 10.1105/tpc.12.9.1667 PMC149077

[CIT0025] Janitza P Ullrich KK Quint M 2012 Toward a comprehensive phylogenetic reconstruction of the evolutionary history of mitogen-activated protein kinases in the plant kingdom. Frontiers in Plant Science 3, 271 23230446 10.3389/fpls.2012.00271 PMC3515877

[CIT0026] Javot H Lauvergeat V Santoni V 2003 Role of a single aquaporin isoform in root water uptake. Plant Cell 15, 509–522 12566588 10.1105/tpc.008888 PMC141217

[CIT0027] Jonak C Okresz L Bögre L Hirt H 2002 Complexity, cross talk and integration of plant MAP kinase signalling. Current Opinion in Plant Biology 5: 415–424 12183180 10.1016/s1369-5266(02)00285-6

[CIT0028] Katsuhara M Koshio K Shibasaka M Hayashi Y Hayakawa T Kasamo K 2003 Over-expression of a barley aquaporin increased the shoot/root ratio and raised salt sensitivity in transgenic rice plants. Plant Cell Physiology 44, 1378–1383 14701933 10.1093/pcp/pcg167

[CIT0029] Kiegerl S Cardinale F Siligan C Gross A Baudouin E Liwosz A Eklöf S Till S Bögre L Hirt H 2000 SIMKK, a mitogen-activated protein kinase (MAPK) kinase, is a specific activator of the salt stress-induced MAPK, SIMK. Plant Cell 12, 2247–2258 11090222 10.1105/tpc.12.11.2247 PMC150171

[CIT0030] Kim JM Woo DH Kim SH Lee SY Park HY Seok HY Chung WS Moon YH 2012 *Arabidopsis* MKKK20 is involved in osmotic stress response via regulation of MPK6 activity. Plant Cell Reports 31, 217–224 21969089 10.1007/s00299-011-1157-0

[CIT0031] Keshet Y Seger R 2010 The MAP Kinase signaling cascades: a system of hundreds of components regulates a diverse array of physiological functions. Methods in Molecular Biology 661, 3–38 20811974 10.1007/978-1-60761-795-2_1

[CIT0032] Komis G Illés P Beck M Šamaj J 2011 Microtubules and mitogen-activated protein kinase signalling. Current Opinion in Plant Biology 14, 650–657 21839668 10.1016/j.pbi.2011.07.008

[CIT0033] Kosetsu K Matsunaga S Nakagami H Colcombet J Sasabe M Soyano T Takahashi Y Hirt H Machida Y 2010 The MAP kinase MPK4 is required for cytokinesis in *Arabidopsis thaliana* . Plant Cell 22, 3778–3790 21098735 10.1105/tpc.110.077164 PMC3015120

[CIT0034] Lee J Rudd JJ Macioszek VK Scheel D 2004 Dynamic changes in the localization of MAP kinase cascade components controlling pathogenesis-related (PR) gene expression during innate immunity in parsley. Journal of Biological Chemistry 279, 22440–22448 15001572 10.1074/jbc.M401099200

[CIT0035] Li GZ Vissers JP Silva JC Golick D Gorenstein MV Geromanos SJ 2009 Database searching and accounting of multiplexed precursor and product ion spectra from the data independent analysis of simple and complex peptide mixtures. Proteomics 9, 1696–1719 19294629 10.1002/pmic.200800564

[CIT0036] Ligterink W Kroj T zur Nieden U Hirt H Scheel D 1997 Receptor-mediated activation of a MAP kinase in pathogen defence of plants. Science 276, 2054–2057 9197271 10.1126/science.276.5321.2054

[CIT0037] Liszkay A Kenk B Schopfer P 2003 Evidence for the involvement of cell wall peroxidase in the generation of hydroxyl radicals mediating extension growth. Planta 217, 658–667 12739149 10.1007/s00425-003-1028-1

[CIT0038] Liu J Ishitani M Halfter U Kim CS Zhu JK 2000 The *Arabidopsis thaliana SOS2* gene encodes a protein kinase that is required for salt tolerance. Proceedings of the National Academy of Sciences, USA 97, 3730–3734 10.1073/pnas.060034197 PMC16308 10725382

[CIT0039] MAPK Group. 2002 Mitogen-activated protein kinase cascades in plants: a new nomenclature. Trends in Plant Science 7, 301–308 12119167 10.1016/s1360-1385(02)02302-6

[CIT0040] Miller G Suzuki N Ciftci-Yilmaz S Mittler R 2010 Reactive oxygen species homeostasis and signalling during drought and salinity stresses. Plant, Cell & Environment 33, 453–467 10.1111/j.1365-3040.2009.02041.x 19712065

[CIT0041] Müller J Beck M Mettbach U Komis G Hause G Menzel D Šamaj J 2010 *Arabidopsis* MPK6 is involved in cell division plane control during early root development, and localizes to the pre-prophase band, phragmoplast, *trans*-Golgi network and plasma membrane. The Plant Journal 61, 234–248 19832943 10.1111/j.1365-313X.2009.04046.x

[CIT0042] Munnik T Ligterink W Meskiene I Calderini O Beyerly J Musgrave A Hirt H 1999 Distinct osmosensing protein kinase pathways are involved in signaling moderate and severe hyperosmotic stress. The Plant Journal 20, 381–388 10607291 10.1046/j.1365-313x.1999.00610.x

[CIT0043] Munns R Tester M 2008 Mechanisms of salinity tolerance. Annual Review of Plant Biology 59, 651–681 10.1146/annurev.arplant.59.032607.092911 18444910

[CIT0044] Nishihama R Ishikawa M Araki S Soyano T Asada T Machida Y 2001 The NPK1 mitogen-activated protein kinase kinase kinase is a regulator of cell plate formation in plant cytokinesis. Genes and Development 15, 352–363 11159915 10.1101/gad.863701 PMC312623

[CIT0045] Passardi F Penel C Dunand C 2004 Performing the paradoxical: how plant peroxidases modify the cell wall. Trends in Plant Science 9, 534–540 15501178 10.1016/j.tplants.2004.09.002

[CIT0046] Pitzschke A Schikora A Hirt H 2009 MAPK cascade signalling networks in plant defence. Current Opinion in Plant Biology 12, 421–426 19608449 10.1016/j.pbi.2009.06.008

[CIT0047] Plumb RS Johnson KA Rainville P Smith BW Wilson ID Castro-Perez JM Nicholson JK 2006 UPLC/MS(E); a new approach for generating molecular fragment information for biomarker structure elucidation. Rapid Communication Mass Spectrometry 20, 1989–1994 10.1002/rcm.2550 16755610

[CIT0048] Prak S Hem S Boudet J Viennois G Sommerer N Rossignol M Maurel C Santoni V 2008 Multiple phosphorylations in the C-terminal tail of plant plasma membrane aquaporins. Molecular & Cellular Proteomics 7, 1019–1030 18234664 10.1074/mcp.M700566-MCP200

[CIT0049] Qiu QS Guo Y Dietrich MA Schumaker KS Zhu JK 2002 Regulation of SOS1, a plasma membrane Na^+^/H^+^ exchanger in *Arabidopsis thaliana*, by SOS2 and SOS3. Proceedings of the National Academy of Sciences, USA 99, 8436–8441 10.1073/pnas.122224699 PMC123085 12034882

[CIT0050] Rasmussen MW Roux M Petersen M Mundy J 2012 MAP kinase cascades in *Arabidopsis* innate immunity. Frontiers in Plant Science 3, 169 22837762 10.3389/fpls.2012.00169 PMC3402898

[CIT0051] Ren D Yang H Zhang S 2002 Cell death mediated by MAPK is associated with hydrogen peroxide production in Arabidopsis. Journal of Biological Chemistry 277, 559–565 11687590 10.1074/jbc.M109495200

[CIT0052] Rodriguez MC Petersen M Mundy J 2010 Mitogen-activated protein kinase signaling in plants. Annual Review of Plant Biology 61, 621–649 10.1146/annurev-arplant-042809-112252 20441529

[CIT0053] Romero-Aranda R Soria T Cuartero J 2001 Tomato plant-water uptake and plant-water relationships under saline growth conditions. Plant Science 160, 265–272 11164598 10.1016/s0168-9452(00)00388-5

[CIT0054] Sasabe M Machida Y 2012 Regulation of organization and function of microtubules by the mitogen-activated protein kinase cascade during plant cytokinesis. Cytoskeleton 69, 913–918 23027702 10.1002/cm.21072

[CIT0055] Shi H Ishitani M Kim C Zhu JK 2000 The *Arabidopsis thaliana* salt tolerance gene *SOS1* encodes a putative Na^+^/H^+^ antiporter. Proceedings of the National Academy of Sciences, USA 97, 6896–6901 10.1073/pnas.120170197 PMC18772 10823923

[CIT0056] Sinha AK Jaggi M Raghuram B Tuteja N 2011 Mitogen-activated protein kinase signaling in plants under abiotic stress. Plant Signaling and Behavior 6, 196–203 21512321 10.4161/psb.6.2.14701 PMC3121978

[CIT0057] Sorkin A von Zastrow M 2002 Signal transduction and endocytosis: close encounters of many kinds. Nature Reviews Molecular Cell Biology 3, 600–614 10.1038/nrm883 12154371

[CIT0058] Staiger CJ Poulter NS Henty JL Franklin-Tong VE Blanchoin L 2010 Regulation of actin dynamics by actin-binding proteins in pollen. Journal of Experimental Botany 61, 1969–1986 20159884 10.1093/jxb/erq012

[CIT0059] Su SH Suarez-Rodriguez MC Krysan P 2007 Genetic interaction and phenotypic analysis of the *Arabidopsis* MAP kinase pathway mutations *mekk1* and *mpk4* suggests signaling pathway complexity. FEBS Letters 581, 3171–3177 17572407 10.1016/j.febslet.2007.05.083

[CIT0060] Šamaj J Ovečka M Hlavačka A 2002 Involvement of the mitogen-activated protein kinase SIMK in regulation of root hair tip-growth. EMBO Journal 21, 3296–3306 12093731 10.1093/emboj/cdf349 PMC126098

[CIT0061] Šamajová O Plíhal O Al-Yousif M Hirt H Šamaj J 2013 *a* Improvement of stress tolerance in plants by genetic manipulation of mitogen-activated protein kinases. Biotechnology Advances 31, 118–128 22198202 10.1016/j.biotechadv.2011.12.002

[CIT0062] Šamajová O Komis G Šamaj J 2013 *b* Emerging topics in the cell biology of mitogen-activated protein kinases. Trends in Plant Science 18, 140–148 23291243 10.1016/j.tplants.2012.11.004

[CIT0063] Takáč T Pechan T Richter H Müller J Eck C Böhm N Obert B Ren H Niehaus K Šamaj J 2011 Proteomics on brefeldin A-treated Arabidopsis roots reveals profilin 2 as a new protein involved in the cross-talk between vesicular trafficking and the actin cytoskeleton. Journal of Proteome Research 10, 488–501 21090759 10.1021/pr100690f

[CIT0064] Teige M Scheikl E Eulgem T Dóczi R Ichimura K Shinozaki K Dangl JL Hirt H 2004 The MKK2 pathway mediates cold and salt stress signaling in *Arabidopsis* . Molecular Cell 15, 141–152 15225555 10.1016/j.molcel.2004.06.023

[CIT0065] Tornroth-Horsefield S Wang Y Hedfalk K Johanson U Karlsson M Tajkhorshid E Neutze R Kjellbom P 2006 Structural mechanism of plant aquaporin gating. Nature 439, 688–694 16340961 10.1038/nature04316

[CIT0066] Uváčková L Škultéty L Bekešová S McClain S Hajduch M 2013 The MS^E^-proteomic analysis of gliadins and glutenins in wheat grain identifies and quantifies proteins associated with celiac disease and baker’s asthma. Journal of Proteomics , 93, 65–73 23268118 10.1016/j.jprot.2012.12.011

[CIT0067] Verslues PE Batelli G Grillo S Agius F Kim Y-S Zhu J Agarwal M Katiyar-Agarwal S Zhu J-K 2007 Interaction of SOS2 with nucleoside diphosphate kinase 2 and catalases reveals a point of connection between salt stress and H_2_O_2_ signaling in *Arabidopsis thaliana* . Molecular and Cellular Biology 27, 7771–7780 17785451 10.1128/MCB.00429-07 PMC2169147

[CIT0068] Volmat V Camps M Arkinstall S Pouyssegur J Lenormand P 2001 The nucleus, a site for signal termination and inactivation of p42/p44 MAP kinases. Journal of Cell Science 114, 3433–3443 11682603 10.1242/jcs.114.19.3433

[CIT0069] Xu J Li Y Wang Y Liu H Lei L Yang H Liu G Ren D 2008 Activation of MAPK kinase 9 induces ethylene and camalexin biosynthesis and enhances sensitivity to salt stress in *Arabidopsis* . Journal of Biological Chemistry 283, 26996–27006 18693252 10.1074/jbc.M801392200

[CIT0070] Yoo SD Cho YH Tena G Xiong Y Sheen J 2008 Dual control of nuclear EIN3 by bifurcate MAPK cascades in C_2_H_4_ signalling. Nature 451, 789–795 18273012 10.1038/nature06543 PMC3488589

[CIT0071] Yu L Nie J Cao C Jin Y Yan M Wang F Liu J Xiao Y Liang Y Zhang W 2010 Phosphatidic acid mediates salt stress response by regulation of MPK6 in *Arabidopsis thaliana* . New Phytologist 188, 762–773 20796215 10.1111/j.1469-8137.2010.03422.x

