# Supplementary material for: Salt-induced subcellular kinase relocation and seedling susceptibility caused by overexpression of Medicago SIMKK in Arabidopsis
Source: J Exp Bot. 2014 Mar 19;65(9):2335–50. doi: 10.1093/jxb/eru115 (PMC4036504; doi:10.1093/jxb/eru115)
Supplement: Supplementary Data [file supp_65_9_2335__index.html]

Salt-induced subcellular kinase relocation and seeding susceptibility caused by overexpression of Medicago SIMKK in Arabidopsis — Supplementary Data 

# Salt-induced subcellular kinase relocation and seedling susceptibility caused by overexpression of *Medicago* SIMKK in *Arabidopsis*

## Supplementary Data

Data files

**Files in this Data Supplement:**

- Supplementary Data - Supplementary Data
- Supplementary Data - Supplementary Data
- Supplementary Data - Supplementary Data
- Supplementary Data - Supplementary Data
