# Supplementary material for: Salt-induced subcellular kinase relocation and seedling susceptibility caused by overexpression of Medicago SIMKK in Arabidopsis
Source: J Exp Bot. 2014 Mar 19;65(9):2335–50. doi: 10.1093/jxb/eru115 (PMC4036504; doi:10.1093/jxb/eru115)
Supplement: Supplementary Data [file supp_eru115_jexbot118018_file001.pdf]

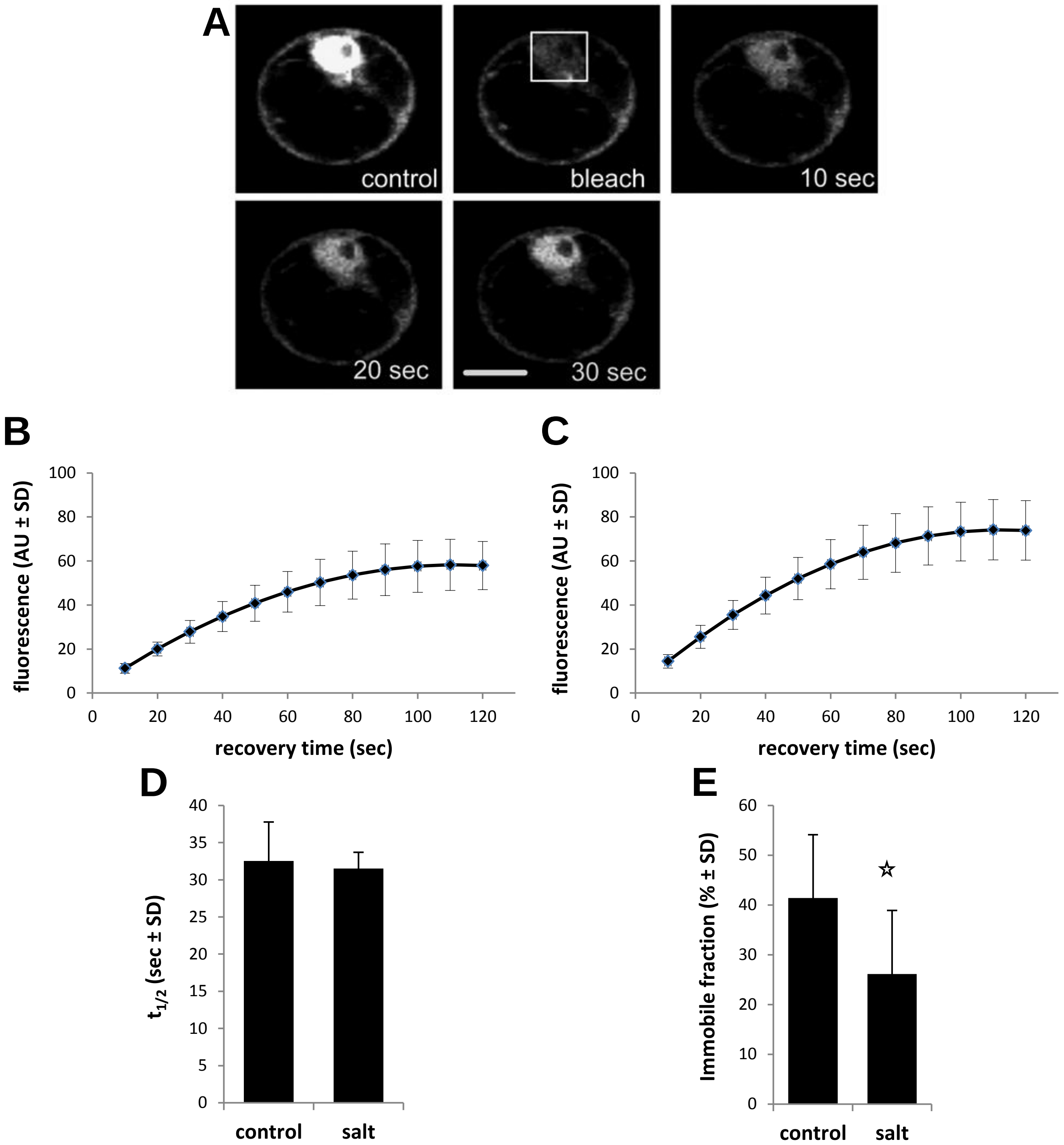

Supplementary Figure S1. Fluorescence recovery after photobleaching (FRAP) analysis of YFP-tagged SIMKK in transiently transformed *Arabidopsis* protoplasts. (A) After selective bleaching of the nucleus (indicated by frame in the middle image) the YFP-tagged SIMKK recovers its nuclear fluorescence suggesting active relocation from the cytoplasm back to the nucleus. Note overall decrease of fluorescence in the cytoplasm with increased time. Recovery time is indicated in seconds. (B-C) Signal recovery in the protoplast nuclei in the control (B) and after treatment with 250 mM NaCl (C). Fluorescence intensities in control and salt experiments are shown in arbitrary units (AU), and normalized to absolute fluorescence intensities in the nuclei before bleaching. (D) Average half-time of signal recovery in the nuclei of control and salt-treated protoplasts (n = 12). (E) Portion of immobile protein fractions calculated from FRAP analysis. Statistically significant difference, as evaluated by Student t-test ($P<0.05$), is indicated by asterisk (n = 12). Three independent experiments were performed. SD = standard deviation. Bar = 10 μm in A.

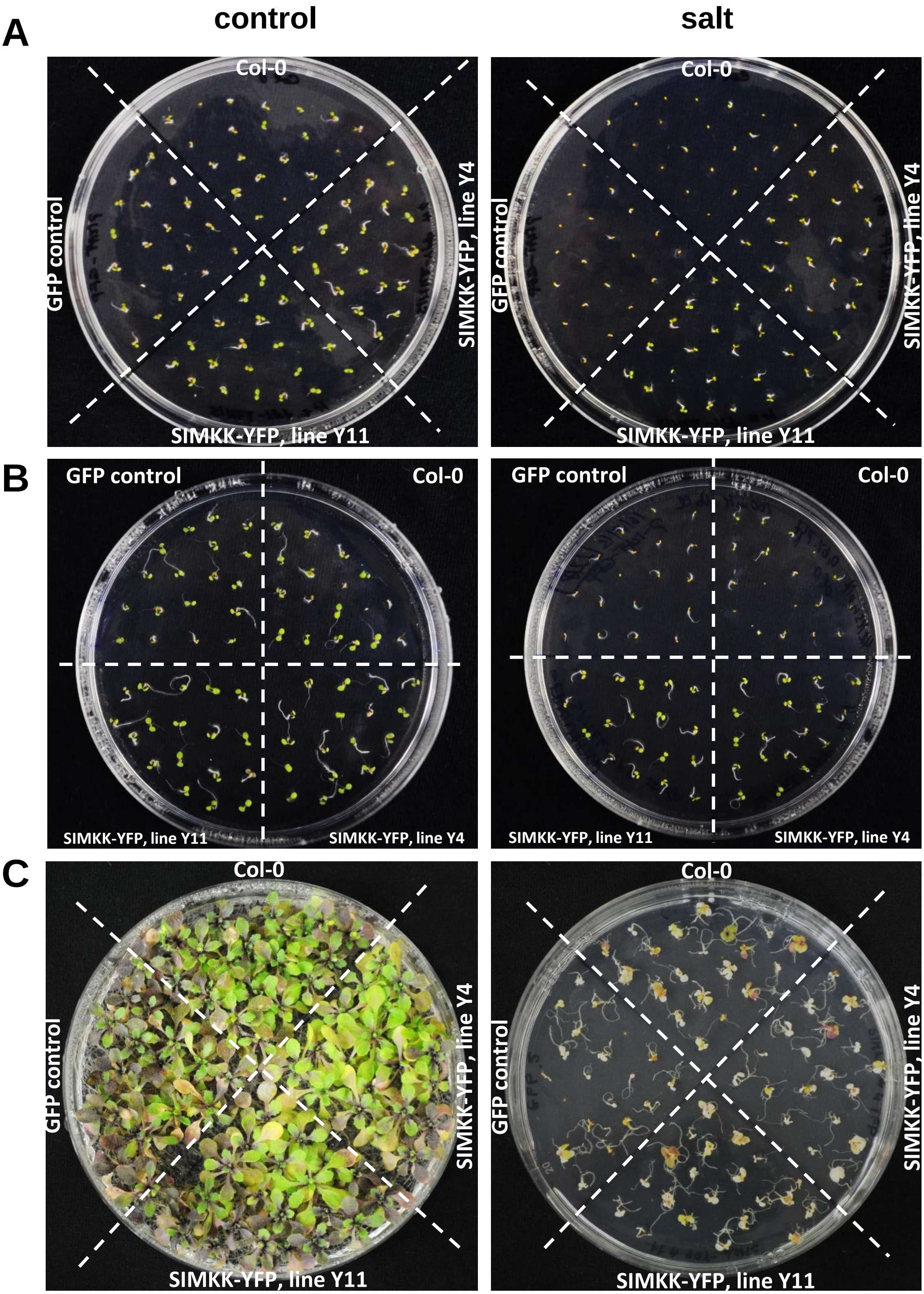

Supplementary Figure S2. Overview of germination test of *Arabidopsis* plants stably transformed with YFP-tagged SIMKK under salt stress. Stages of seed germination of wild type Col-0, stably transformed line with GFP alone (line G5) and transformed lines expressing YFP-tagged SIMKK (lines Y4 and Y11) on control and 100 mM NaCl-containing media. Plates were photographed 2 days (A), 3 days (B) and 23 days (C) after sowing.

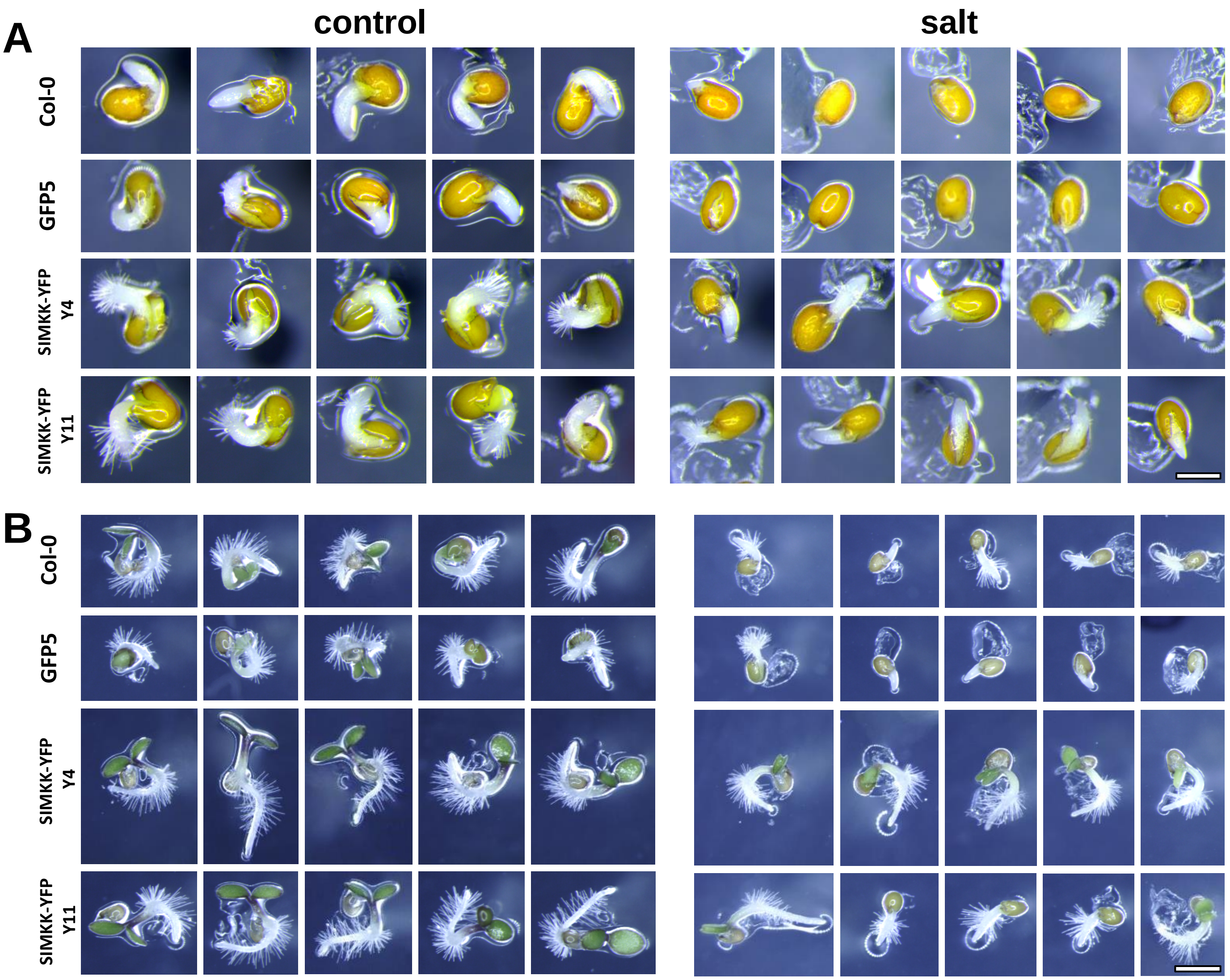

Supplementary Figure S3. Details of germination test of *Arabidopsis* plants stably transformed with YFP-tagged SIMKK under salt stress. Stages of seed germination of wild type Col-0, stably transformed line with GFP alone (line G5) and transformed lines overexpressing YFP-tagged SIMKK (lines Y4 and Y11) in control and 100 mM NaCl-containing media. (A) One day after sowing, (B) two days after sowing. Bar = 0.5 mm in A and 1 mm in B.
